# Economic evaluation of interventions for treatment-resistant depression: A systematic review

**DOI:** 10.3389/fpsyt.2023.1056210

**Published:** 2023-02-16

**Authors:** Laura A. Hannah, Cathy M. Walsh, Louise Jopling, Jesus Perez, Rudolf N. Cardinal, Rory A. Cameron

**Affiliations:** ^1^Applied Research Collaboration East of England, National Institute for Health and Care Research, Cambridge, United Kingdom; ^2^Cambridgeshire and Peterborough NHS Foundation Trust, Cambridge, United Kingdom; ^3^Eastern Academic Health Science Network, Cambridge, United Kingdom; ^4^Department of Psychiatry, University of Cambridge, Cambridge, United Kingdom; ^5^Norwich Medical School, University of East Anglia, Norwich, United Kingdom; ^6^Psychiatry Unit, Department of Medicine, Institute of Biomedical Research of Salamanca, University of Salamanca, Salamanca, Spain

**Keywords:** economic evaluation, health economics, treatment-resistant depression, persistent depression, values based commissioning

## Abstract

**Background:**

The extraordinarily high prevalence of treatment-resistant depression (TRD), coupled with its high economic burden to both healthcare systems and society, underscore how critical it is that resources are managed optimally to address the significant challenge it presents.

**Objective:**

To review the literature on economic evaluation in TRD systematically, with the aim of informing future studies by identifying key challenges specific to the area, and highlighting good practices.

**Methods:**

A systematic literature search across seven electronic databases was conducted to identify both within-trial and model-based economic evaluations in TRD. Quality of reporting and study design was assessed using the Consensus Health Economic Criteria (CHEC). A narrative synthesis was conducted.

**Results:**

We identified 31 evaluations, including 11 conducted alongside a clinical trial and 20 model-based evaluations. There was considerable heterogeneity in the definition of treatment-resistant depression, although with a trend for more recent studies to use a definition of inadequate response to two or more antidepressive treatments. A broad range of interventions were considered, including non-pharmacological neuromodulation, pharmacological, psychological, and service-level interventions. Study quality as assessed by CHEC was generally high. Frequently poorly reported items related to discussion of ethical and distributional issues, and model validation. Most evaluations considered comparable core clinical outcomes – encompassing remission, response, and relapse. There was good agreement on the definitions and thresholds for these outcomes, and a relatively small pool of outcome measures were used. Resource criteria used to inform the estimation of direct costs, were reasonably uniform. Predominantly, however, there was a high level of heterogeneity in terms of evaluation design and sophistication, quality of evidence used (particularly health state utility data), time horizon, population considered, and cost perspective.

**Conclusion:**

Economic evidence for interventions in TRD is underdeveloped, particularly so for service-level interventions. Where evidence does exist, it is hampered by inconsistency in study design, methodological quality, and availability of high quality long-term outcomes evidence. This review identifies a number of key considerations and challenges for the design of future economic evaluations. Recommendations for research and suggestions for good practice are made.

**Systematic review registration:**

https://www.crd.york.ac.uk/prospero/display_record.php?RecordID=259848&VersionID=1542096, identifier CRD42021259848.

## 1. Introduction

Major depressive disorder (MDD) affects approximately 5% of the global population and continues to be a major contributor to the overall global burden of disease (1). There is strong evidence that the prevalence of MDD is increasing (2), with the COVID-19 pandemic driving prevalence rates yet higher. Response to the global health crisis and strategies used to prevent the spread of the virus, constructed an environment whereby factors contributing to MDD onset and reoccurrence were exacerbated; contributing to a 28% rise in global prevalence rates (3). Since many of these factors persist (including, but not restricted to: constrained healthcare resources; widened socioeconomic inequality; social isolation; neuropsychiatric sequelae), this trend is not expected to retreat in the near-term (4, 5).

Response to treatment of MDD varies, with many patients requiring more than one treatment step (6). A third of patients do not report improved symptoms despite multiple interventions, resulting in a persistent form of depression commonly described as “treatment-resistant depression” (TRD) (7). Defining TRD is problematic, since failure to respond to treatment “exists on a continuum” (8). A recent review found that while the most widely used definition for TRD was a failure to respond to two or more treatments at an adequate dose and duration, only 19% of recent interventional TRD studies were consistent with that definition (9).

Reflecting this heterogeneity in classification of TRD (10), and indeed in the patient population (11), no single treatment pathway exists, although a stepped-care approach is recommended. Such a model aims to address scarce treatment resources by ensuring that the most effective, least restrictive treatments (in terms of both healthcare resources, and patient convenience), are delivered first, with patients “stepped up” to more intensive treatments as needed (12). Recent UK National Institute for Health and Care Excellence (NICE) guidelines (13) advocate starting treatment for moderate to severe MDD with psychological interventions, such as cognitive–behavioural therapy (CBT), combined with an antidepressant. Where symptoms persist after 4–6 weeks, additional treatments and referral to secondary/specialist mental health services should be considered. Further treatments may include increasing the antidepressant dose, switching to another antidepressant medication of the same or different class, switching to another psychological therapy, adding a second-generation antipsychotic or lithium, or augmenting with electroconvulsive therapy (ECT), lamotrigine, or triiodothyronine. Other treatment options include repetitive transcranial magnetic stimulation (rTMS) and implanted vagus nerve stimulation.

Despite this diverse armamentarium, there remains a high unmet need for new and cost-effective interventions (14, 15). Unfortunately, the condition is highly recurrent—80% of TRD patients experience relapse within a year of remission and the probability of sustained remission over 10 years is just 40% (16). A well-established body of evidence has demonstrated that increasing treatment resistance is associated with poorer health-related quality of life (HRQoL) (8), increased direct medical costs (8, 17, 18), and indirect costs to society attributed to impairments in work productivity and activity (15, 19), and social care demands (20).

Against a background of increasingly constrained healthcare budgets, it is important that decision makers consider not only clinical effectiveness, but the economic evidence for interventions, in order to identify and prioritize those that make the best use of available resources (21). Previous systematic reviews of economic evaluations of interventions for MDD have reported considerable uncertainty in their findings due to inconsistent methodological quality and results (22), and highlighted a lack of evidence and good quality data in TRD (23, 24). Johnston et al. (8) reviewed the literature on the economic burden of TRD, and found significant methodological and population disparities, highlighting heterogeneity in defining TRD, the outcomes measured, and the health state utility values reported.

The aim of this review is to appraise the existing evidence and methods used in economic evaluations of interventions for TRD, and to make best-practice recommendations to inform the development of future evaluations. Promoting consistency in evaluation methodology will improve confidence when making resource allocation decisions, and increase the likelihood that promising interventions receive appropriate funding or support.

## 2. Concepts in health economic evaluation

### 2.1. Type of economic evaluation

A “full” health economic evaluation compares both the costs and the consequences of alternative courses of actions (25). The output of the evaluation is (typically) an incremental cost-effectiveness ratio (ICER) (26). Depending on the outcome measure used, economic evaluations may be classified as: cost-effectiveness analyses (CEA), when a clinical outcome measure is used; cost benefit analyses (CBA), when outcomes are valued in monetary terms; cost utility analysis (CUA), when health outcomes are valued as health state utilities to derive quality adjusted life years; cost consequence analysis (CCA), where multiple outcomes not easily summarized in a single summary measure are presented in a disaggregated format; and cost minimisation analysis (CMA), which assumes that the outcomes from the alternatives under consideration are equivalent (27).

### 2.2. Health state utilities

Health state utilities are used to represent the “value” of different health states, based on a surveyed population’s strength preferences for those health states. Utilities are conventionally scaled between 0 and 1, with 1 representing the value of perfect health and 0 representing the valuation of death (28). Some systems allow a negative utility value, whereby very poor health states may be valued as less preferable than death. When measured over time, utilities may be used to derive the quality adjusted life years (QALYs) associated with living in a particular health state (29).

### 2.3. Perspective

The perspective of the evaluation refers to the breadth of costs and benefits that are to be considered in the evaluation. Most commonly, the perspective of the healthcare provider or payer is adopted; at the broadest, a “societal” perspective reflects a comprehensive range of social opportunity costs associated with the alternatives under consideration (30). Where significant opportunity costs exist outside the healthcare system, for example in public health interventions, a broad perspective is advised, and there is growing support for such a broad perspective to be used in mental health economic evaluation (21). The 2016 Second Panel on Cost Effectiveness in Health and Medicine recommends analysts adopt a comprehensive approach, reporting separately both healthcare sector and societal perspectives (31). The Panel further recommends the societal perspective report costs and consequences in a comprehensive “impact inventory,” and where possible, that non-health consequences are quantified and valued (31). While methodological guidance on choice of perspective varies by jurisdiction, it is generally agreed that the choice should be explicitly stated and determined by the study sponsor (and any stakeholders identified by the sponsor) (32).

### 2.4. Time horizon

The time horizon refers to the period over which the costs and benefits of the evaluation are captured. Choice of time horizon is influenced by the nature of the condition and intervention under evaluation, and the framework and purpose of the analysis. Ideally, the time horizon for economic evaluations should be sufficiently long to capture relevant differences in costs and outcomes between the comparators; for many interventions, this requires a lifetime horizon (33, 34). Where extrapolated data are used, this is likely to require the analyst to make assumptions about the continued efficacy of the interventions (35).

### 2.5. Study design

Economic evaluations of health care interventions typically follow one of two study designs: “within-trial” evaluations, where the costs and benefits of alternative courses of action are collected alongside clinical data in interventional clinical studies; and those that use decision analytic models.

#### 2.5.1. Within trial designs

Within-trial evaluations have the advantage that the costs and consequences of the interventions under investigation are measured directly, but are constrained by the follow-up period, frequently precluding assessment of long-term cost effectiveness (36). Extrapolation may be possible using survival analysis models, though this approach requires related long-term data on costs, benefits and complications of the interventions (37).

Sample size and power estimates for trials are most commonly based on the primary clinical outcome. Owing to the tendency of cost variables to have much greater variance than clinical outcomes, trial-based economic evaluations are often underpowered to detect statistically significant differences in cost (38). Accordingly, health economic evaluations assess the probability of cost effectiveness against a certain threshold of willingness-to-pay (WTP), rather than employing statistical hypothesis tests concerning cost effectiveness (37). Typically, probability of cost-effectiveness is assessed against a range of WTP values, and is represented in a cost effectiveness acceptability curve, representing from the joint distribution of incremental costs and effects (37, 39). Most commonly, this distribution is estimated using non-parametric bootstrapping to address sampling uncertainty (39).

Best practice guidelines encourage the use of robust methods to address missing data, since exclusion of cases with missing or censored data may introduce bias (33). While several approaches may be adopted for handling missing data, (including complete case analysis, single imputation and inverse probability weighting), the use of multiple imputation models are usually recommended (40), although this approach may be contested when evaluating data with a high degree of missingness (41).

Combining methods for addressing sampling uncertainty and those for addressing missing data, however, is non-trivial and presents challenges both practical challenges (e.g., computational intensivity), and statistical challenges (e.g., the artificial reduction of sampling uncertainty through imputation) (33, 42). There is a need for further research in this area, as currently no consensus exists for best practice approaches (41).

#### 2.5.2. Decision analytic model designs

Decision analytic models may be used to extrapolate the findings of clinical trial over a longer “time horizon,” or to a different population, or may be used to compare interventions for which no head-to-head trials have yet been conducted. Economic models are mathematical abstractions of the real world: analysts will work with subject-matter experts to conceptualize a specific structure, the contingent assumptions, and required input parameters (43). The models describe the probability of specific outcomes following an intervention, with the costs and benefits of each outcome having an associated value. The expected value of that intervention is expressed as the sum of values for each outcome, weighted by the probability of the outcome (43).

Three approaches are commonly used in decision analytic economic evaluation models. The decision tree is a simple but widely used approach used to evaluate short-term prognoses, represented by a series of pathways (44). Markov cohort models may be used to evaluate outcomes over a lifetime horizon, and typically model a homogeneous population transitioning through a series of “health states.” Transitions are modeled in a series of cycles (of a length defined by the analyst); a key property (and frequently a problematic assumption) of Markov models is that no “memory” of the events of previous cycles is retained through each transition (45). Individual-level microsimulation models, which may take the same form as a Markov model, facilitate modeling of a heterogeneous population, and the impact of past events (e.g., number of treatment failures, or adverse events), on prognosis (46).

Analogous to bootstrapping in within-trial evaluations, parametric methods (e.g., Monte Carlo simulation), are recommended to generate sampling distributions of joint mean cost and efficacy estimates (47).

## 3. Methods

This systematic review follows guidance provided by the Preferred Reporting Items for Systematic Reviews and Meta-Analyses (PRISMA) group (48). The study protocol was registered with the International Prospective Register of Systematic Reviews (PROSPERO; registration CRD42021259848).

### 3.1. Eligibility criteria

Predefined inclusion criteria, defined by the Population, Intervention, Comparator, Outcome, Study type (PICOS) framework (Table 1) were used to determine study selection. Evaluations were included if the author defined the population as “persistent/treatment-resistant/treatment-refractory depression,” within adult populations (i.e., individuals aged at least 18 years). Any intervention, across all treatment settings (primary, secondary, and/or community care), relating to the treatment or management of TRD were eligible. Evaluations were excluded if there was no comparator, where comparators could include placebo, an alternative to standard treatment, or treatment as usual.

**TABLE 1:** Review inclusion criteria.

Criteria	Notes
Population	Adults with treatment-resistant depression
Intervention	Any intervention for the management of TRD
Comparator	Any intervention for the management of TRD
Outcome	Incremental changes in costs and consequences
Study types	Full economic evaluations: cost-utility analyses (CUA); cost-effectiveness analyses (CEA); cost-benefit analyses (CBA); cost-minimization analyses (CMA); and cost-consequence analyses (CCA). Model and trial-based studies included
Language	English
Time frame	Any
Exclusion	No comparator No consideration of incremental Δcost and Δconsequences

Evaluation types included any “full” economic evaluation that considered incremental changes across both costs and consequences (CUA, CEA, CBA, CMA, and CCA).

Included evaluations were required to be full-length, peer-reviewed interventional, observational, or modeling reports in journal or Health Technology Authority (HTA) publications in the English language. No date restrictions were imposed. Additionally, bibliographies of systematic reviews were examined to identify further potentially relevant evaluations; however, such reviews themselves were excluded.

### 3.2. Information sources and search strategy

Searches across seven electronic databases (MEDLINE; Embase; Cochrane Database of Systematic Reviews; NHS Economic Evaluation Database; Health Technology Assessment database; CINAHL; and PsycINFO) were conducted from inception to 19th May 2021. Searches used two primary concepts (population AND study type), described by Medical Subject Heading (MeSH) and free text search terms. Search terms were refined using Boolean, truncation and adjacency operators. Full search strategies are available in Supplementary Table 1.

### 3.3. Study selection

Records identified in the search strategy were uploaded to the Rayyan platform,^1^ for de-duplication and screening. All papers were examined against the PICOS inclusion and exclusion criteria independently by two reviewers (RC and LH) in a two-stage process; title and abstract followed by full-text screening. Reviewers discussed conflicts after each phase and a consensus was reached.

### 3.4. Data extraction and quality assessment

Key study information was extracted using a pre-defined spreadsheet in Microsoft Excel. Two reviewers (RC and LH) conducted data extraction with a 30% overlap in evaluations. Level of agreement between the overlapping extractions were compared and discussed. Disagreements regarding the content of the extraction fields were resolved through discussion. Data extraction fields included: evaluation details (publication type, setting, objectives); population; general evaluation characteristics (type of intervention and controls, perspective, type of evaluation used, study design, time horizon and reference year); resource use and costs (type of category and costs, data source, and methods used to calculate costs); outcomes (primary clinical outcomes, other clinical outcomes, economic outcomes, and data source for outcomes); economic evaluation results (incremental costs and effects, summary measure of benefits, cost effectiveness results, analyses of uncertainty, and author’s conclusions); and model-based evaluation characteristics (model type, model structure and assumptions, rationale for model type and structure, consideration of population heterogeneity).

The Consensus on Health Economic Criteria (CHEC) was used for quality-of-reporting assessment (49). The 19-item CHEC is recommended for systematic reviews that incorporate both trial-based and model-based economic evaluations (50). Additional items related to model conceptualization were included in the assessment: rationale for model type; rationale for model structure; whether sufficient information was provided to reproduce the model. These items have not been validated, but were informed by items within the “Phillips checklist” for decision analytic models (51).

### 3.5. Analysis

Evaluation characteristics, design, key cost and outcome parameters, and results were synthesized in summary tables and a narrative synthesis approach was used to describe common features and key differences amongst identified economic evaluations.

## 4. Results

### 4.1. Search results and evaluation selection

**FIGURE 1:**
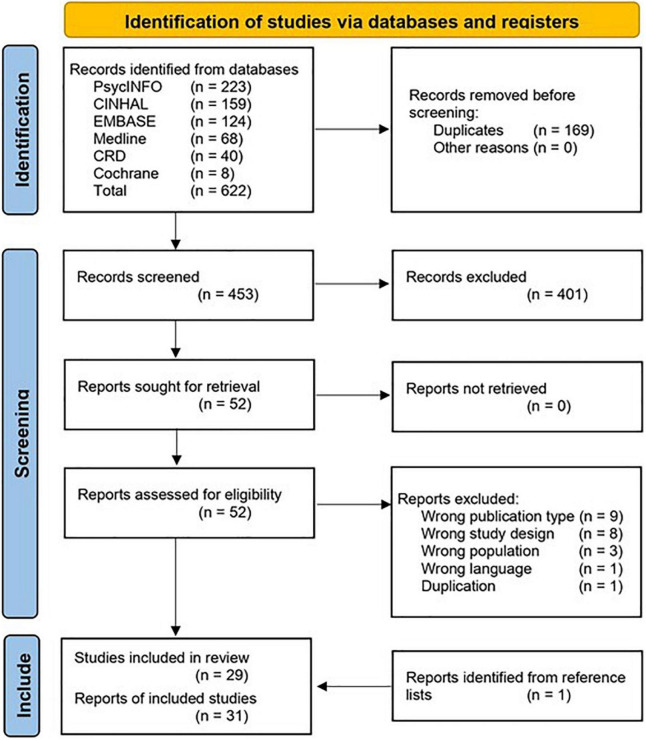
PRISMA flow diagram of study identification, adapted from (48).

The evaluation selection process is summarized in Figure 1. A total of 539 records were identified through the literature searches, and one more was found through screening reference lists (52). After removing 85 duplicates, 400 records clearly failed to meet the inclusion criteria, or met at least one exclusion criterion, leaving 52 for full-text screening. Of these, 31 satisfied the inclusion criteria and were selected for review (52–82).

### 4.2. Summary of included evaluations

Key characteristics of the included economic evaluations are provided in Tables 2A–D. The interventions considered are categorized into four groups:

**TABLE 2A:** Characteristics of economic evaluations of non-pharmacological neuromodulation interventions.

References, country	TRD population/Definition	Comparators	Evaluation type^a^	Study design	Perspective(s)^a^	Time horizon
Nguyen and Gordon (62) Australia	Inadequate response to ≥2 AD treatments	rTMS vs TAU (pharmacotherapy)	CUA	Model	Health system	36 months
Simpson et al. (66) USA	Inadequate response to 1-4 AD treatment	TMS vs TAU (pharmacotherapy) vs Sham TMS	CUA	Model	Health system Societal	12 months
Ross et al. (64) USA	Inadequate response to ≥2 AD treatments	ECT at different therapy lines vs TAU (pharmacotherapy)	CUA	Model	Health system	48 months
McDonald et al. (72) USA	Geriatric TRD on maintenance treatment TRD not explicitly defined	ECT vs TAU (pharmacotherapy)	CCA	Within-trial (non-randomized)	Health system	12 months
Fitzgibbon et al. (56) Canada	Not explicitly defined	rTMS vs ECT vs rTMS + ECT stepped pathway	CUA	Model	Societal	Lifetime
Ghiasvand et al. (57) Iran	Not explicitly defined	rTMS vs ECT	CEA CUA	Model	Health system	7 months
Health Quality Ontario (58) Canada	Inadequate response to ≥ 2 AD treatments	rTMS vs ECT vs sham rTMS	CUA	Model	Health system	6 months
Unit University of Calgary (59) Canada	Inadequate response to ≥2 AD treatments	rTMS vs ECT	CUA	Model	Health system	1.5 months
Kozel et al. (60) USA	Not explicitly defined	rTMS vs ECT vs rTMS + ECT stepped pathway	CUA	Model	Societal	12 months
Vallejo-Torres et al. (67) Spain	Not explicitly defined	ECT vs rTMS vs rTMS + ECT stepped pathway	CUA	Model	Health system	12 months
Xie et al. (69) Canada	Inadequate response to ≥2 AD treatments	rTMS vs ECT vs sham rTMS + TAU	CUA	Model	Health system	6 months
Galletly et al. (52) Australia	Inadequate response to ≥2 AD treatments	rTMS vs ECT	CUA	Model	Health system	36 months
Zhao et al. (71) Singapore	Not explicitly defined	rTMS vs ECT	CUA	Model	Societal	12 months
Knapp et al. (75) UK	Not explicitly defined	rTMS vs ECT	CEA CUA	Within-trial	Health system Societal	6 months

^a^Primary analysis shown first.

**TABLE 2B:** Characteristics of economic evaluations of pharmacological interventions.

References, country	TRD population/Definition	Comparators	Evaluation type^a^	Study design	Perspective(s)^a^	Time horizon
Atlas et al. (53) USA	Inadequate response to ≥2 AD treatments	Esketamine + Antidepressant vs Antidepressant	CUA CEA	Model	Health system Societal	Lifetime
Desai et al. (54) USA	Inadequate response to ≥2 AD treatments	Esketamine + Antidepressant vs Placebo + Antidepressant	CEA	Model	Health system (4 alternative payer perspectives)	12 months
Ross and Soeteman (65) USA	Inadequate response to ≥2 AD treatments	Esketamine + TAU (pharmacotherapy) vs TAU	CUA	Model	Health system Societal	60 months
Edwards et al. (55) UK	Inadequate response to ≥2 AD treatments	AAP + Antidepressant vs Lithium + Antidepressant	CUA	Model	Health system	12 months
Malone (61) USA	Inadequate response to single AD treatment	Antidepressants (escitalopram, paroxetine CR, sertraline, venlafaxine) Generic SSRIs	CEA	Model	Health system	6 months
Olgiati et al. (63) USA	Inadequate response to single AD treatment	Sequenced treatment (switch/augment following citalopram non-response) vs Continued citalopram	CUA	Model	Health system	6 months
Wang et al. (68) UK	Inadequate response to ≥2 AD treatments	Hypothetical monotherapy vs SSRI + AAP	CUA	Model	Health system	12 months
Young et al. (70) UK	Inadequate response to ≥2 AD treatments	Vortioxetine vs SSRIs vs SNRIs vs agomelatine	CUA	Model	Health system	24 months
Kessler et al. (74) UK	Inadequate response to single AD treatment	mirtazapine vs placebo + TAU (pharmacotherapy)	CUA CEA	Within-trial	Health system Societal	12 months

^a^Primary analysis shown first. SSRI, selective serotonin reuptake inhibitor; SNRI, serotonin/noradrenaline reuptake inhibitor; CR, controlled release; TAU, treatment as usual; AAP, atypical antipsychotic.

**TABLE 2C:** Characteristics of economic evaluations of psychological interventions.

References, country	TRD population/Definition	Comparators	Evaluation type^a^	Study design	Perspective(s)^a^	Time horizon
Hollinghurst et al. (73) UK	Inadequate response to single AD treatment	CBT + TAU (usual clinical care for primary care TRD patients) vs TAU	CUA CCA	Within-trial	Health system Societal	12 months
Wiles et al. (82) UK	Inadequate response to ≥6 weeks AD treatment	CBT + TAU (usual clinical care for primary care TRD patients) vs TAU	CUA	Within-trial	Health system	Up to 46 months
Scott et al. (78) UK	Current residual symptoms of ≥8 weeks’ duration following and MDD episode between last 2–18 months	CBT + TAU (usual clinical care) vs TAU	CEA	Within-trial	Health system	17 months
Town et al. (81) Canada	Inadequate response to ≥6 weeks AD treatment	ISTDP + TAU (usual clinical care for secondary care TRD patients) vs TAU	CUA CEA	Within-trial	Health system	18 months
Shearer et al. (79) UK	MDD lasting ≥ 2 years or ≥2 MDD episodes with inadequate response to ≥ 6 weeks AD treatment	RO-DBT + TAU (usual clinical care for secondary care TRD patients) vs TAU	CUA CEA	Within-trial	Health system	12 months
Lynch et al. (76) UK	Inadequate response to ≥2 AD treatments	RO-DBT + TAU (usual clinical care) vs TAU	CUA	Within-trial	Health system Societal	18 months

^a^Primary analysis shown first. AD, antidepressant (drug).

**TABLE 2D:** Characteristics of economic evaluations of service-level interventions.

References, country	TRD population/Definition	Comparators	Evaluation type^a^	Study design	Perspective(s)^a^	Time horizon
Morriss et al. (77) UK	Inadequate response to ≥6 months secondary mental healthcare	Specialist depression service (SDS) vs TAU (usual clinical care for secondary care TRD patients)	CUA	Within-trial	Health system	18 months
Simon et al. (80) USA	≥2 depressive episodes	Collaborative care program vs TAU (usual clinical care for primary care TRD patients)	CEA	Within-trial	Health system	6 months.

^a^Primary analysis shown first.

(a) Non-pharmacological neuromodulation (hereafter referred to as “neuromodulation”), *n* = 14:

1. Repetitive transcranial magnetic stimulation (rTMS) versus Electroconvulsive therapy (ECT), *n* = 10.
2. ECT versus treatment as usual (TAU), *n* = 2.
3. rTMS versus TAU, *n* = 2.

(b) Pharmacological agents *n* = 9:

1. Adjunctive esketamine versus TAU or placebo and TAU, *n* = 3.
2. Adjunctive atypical antipsychotics versus lithium or hypothetical monotherapy, *n* = 2.
3. Mirtazapine versus TAU, *n* = 1.
4. Multiple alternative antidepressant therapies, *n* = 3.

(c) Psychological therapies *n* = 6:

1. Cognitive-behavioral therapy (CBT]) versus TAU, *n* = 3.
2. Radically open dialectical behavior therapy [RO-DBT] versus TAU, *n* = 2.
3. Intensive short-term dynamic psychotherapy [ISTDP] versus TAU, *n* = 1.

(d) Service-level interventions versus TAU, *n* = 2:

The 31 evaluations, relate to 29 unique studies, with multiple economic evaluations included for two studies: a trial comparing the cost effectiveness of rTMS and ECT; (58, 69) and a trial of CBT as an adjunct to pharmacotherapy (73, 82). Of the 31 evaluations included, 11 were trial-based (predominantly psychological [*n* = 6], or service-level [*n* = 2] interventions), and 20 were model-based (predominantly non-pharmacological neuromodulation [*n* = 12] or pharmacological [*n* = 8] interventions). Twenty-four of the evaluations adopted a cost-utility analysis (CUA) as their primary analytical approach, six used cost-effectiveness analysis (CEA), and one adopted a cost-consequence analysis (CCA) as the primary method of evaluation. Six evaluations used multiple analytical approaches. The median time horizon was 1 year, eight evaluations used a time horizon of less than a year, and only two evaluations considered a lifetime horizon. The primary analysis for most evaluations (*n* = 28) considered costs from a healthcare provider perspective, three evaluations considered a (partial) societal perspective, and seven presented both societal and healthcare provider perspectives. The evaluations came almost exclusively from high income countries (UK [*n* = 11]; US [*n* = 10]; Canada [*n* = 5]; Australia [*n* = 2]; Singapore [*n* = 1]; Spain [*n* = 1]), with a single evaluation from Iran) (57).

### 4.3. Quality of reporting assessment

Quality of reporting of the evaluations was predominantly high; the range of fulfilled CHEC criteria across the evaluations fell between 47 and 100%, with an average of 83% of criteria fulfilled. Five evaluations met all criteria from the CHEC-list, and only two evaluations fulfilled fewer than 60% of the criteria (57, 61). The lowest-scoring items from the checklist were: discussion of ethical and distributional issues (45% of evaluations); reporting of structural assumptions and validation methods of models (55% of relevant evaluations); consideration of the generalizability of the results (61% of evaluations). Additional items used to evaluate reporting of conceptualization of model-based evaluations were less well reported: only 15% provided a rationale for choice of model type, and 55% provided a rationale for the model structure. Results of the quality assessment are presented in Supplementary Table 2.

### 4.4. TRD population

There was variation in patient populations considered by the included evaluations, reflecting a lack of consensus on the definition of TRD (83). Most commonly, treatment resistance was defined as a failure to achieve an adequate response to antidepressive treatment (*n* = 24), with half of these specifying a requirement for failure of at least two lines of therapy. Three evaluations used a definition based on the number of previous episodes, or duration of the current episode, and four evaluations did not clearly define treatment-resistant depression or the studied population. At baseline, the populations considered were typically severely depressed, however, severity was not well defined in most model-based studies and had to be intuited from the utility values reported.

### 4.5. Effects

#### 4.5.1. Clinical outcomes

Trial-based evaluations tended to use either response (*n* = 4) or change in depressive symptoms (*n* = 5) as their primary clinical outcome, with only one evaluation using remission (in addition to change in depressive symptoms). Other outcomes included relapse (*n* = 2), and depression-free days. Model-based evaluations tended to include both response and remission (*n* = 13), with five evaluations modeling remission only, one evaluation modeling response, and one modeling change in depressive symptoms.

In trial-based evaluations, the most common outcome measure was the Hamilton Depression Rating Scale (HAM-D, *n* = 6), followed by the Beck Depression Inventory II (BDI-II, *n* = 3). Other measures included the Montgomery–Åsberg Depression Rating Scale (MADRS, *n* = 1), Symptom Checklist–90 (SCL-90, *n* = 1), Beck Depression Inventory (BDI, *n* = 1), and the Global Assessment of Functioning scale (GAF, *n* = 1). Model-based evaluations typically synthesized outcomes from multiple sources, where outcomes may have been measured using several scales, though most frequently mentioned scales included the MADRS (*n* = 10) and the HAM-D (*n* = 9).

Response was typically defined as an improvement of ≥50% from baseline against the scales used, however, there was some variation in the scores used to define remission (HAM-D: <7 [*n* = 1]; ≤7 [*n* = 6]; ≤8 [*n* = 3]; MADRS:≤10 [*n* = 7]; <12 [*n* = 1]; ≤12 [*n* = 2]). In doing so, most evaluations diverged from broadly accepted cut-offs for defining remission: ≤7 for HAM-D (84), and <10 for MADRS (85, 86).

#### 4.5.2. Health economic outcomes

All CUA evaluations used quality-adjusted life years (QALYs) as the primary economic outcome measure. Of these, for most (*n* = 20), utility values underpinning QALY estimates were derived from the EuroQol 5-Dimension 3-Level Health Scale (EQ-5D-3L), the most widely recommended measure of health-related quality of life by HTA authorities globally (87). Other measures included the EuroQol 5-Dimension 5-Level Health Scale (EQ-5D-5L, *n* = 1) (88), the Short-Form Six-Dimension health index (SF-6D, *n* = 4) (73, 89), McSad (*n* = 2) (90), Health Utilities Index 3 (HUI3, *n* = 1) (91), and a vignette-based valuation of various levels of severity of MDD (*n* = 4) (92).

Of the nine evaluations that used a CEA approach (including four as secondary analyses), the most common economic outcome measures were cost per unit change in depression scale rating (*n* = 4), and cost per remitter (*n* = 3). Alternative outcomes included cost per relapse prevented (78), and cost per depression-free day (80).

The single CCA evaluation used maintenance of response and maintenance of relapse as outcome measures (72). All clinical and health economic outcome measures used are summarized in Supplementary Table 3.

### 4.6. Resource use and cost data

Generally, costs were well reported, although several evaluations only reported costs at an aggregate level (53, 57, 60, 61, 64, 65). Trial-based evaluations primarily used self-report questionnaires to collect resource use data (*n* = 8), but also relied on registry or hospital chart data (*n* = 4), and claims databases (*n* = 2). Most model-based evaluations drew data from the literature (*n* = 10), claims databases (*n* = 6), or registry or hospital chart data (*n* = 5).

Direct costs reported for all evaluations included treatment costs, with most also including outpatient (*n* = 27) and inpatient costs (*n* = 26); only three evaluations explicitly included costs for adverse events (AEs). Reported detail concerning assumptions and methods for estimating attribution of capital equipment costs for neuromodulation interventions varied considerably. Indirect costs were considered by the ten evaluations that considered a broader cost perspective, but the scope of items collected varied considerably. Most (*n* = 9) considered productivity (in most cases measuring only absenteeism, although one also measured presenteeism) (76) others additionally considered out-of-pocket payments (*n* = 4), informal care (*n* = 4), formal societal or community care (*n* = 3), or transport (*n* = 3), but no two evaluations included the same set of indirect cost measures.

### 4.7. Modeling approaches and scope

**FIGURE 2:**
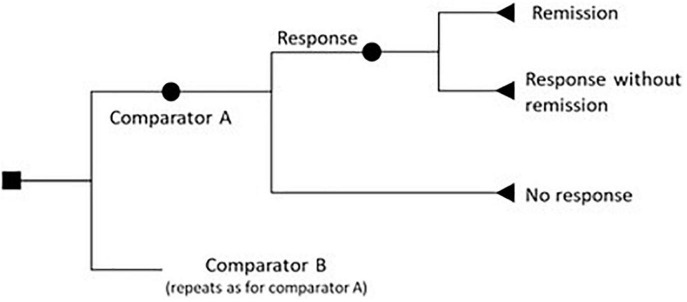
Generic decision tree structure for economic evaluations in TRD.

The details of the 20 models appraised are given in Tables 3A, B. Six evaluations used a decision tree approach, the majority of which (*n* = 5) were evaluations of non-pharmacological neuromodulation interventions, while the sixth compared novel selective serotonin reuptake inhibitors (SSRIs)/serotonin and norepinephrine reuptake inhibitors (SNRIs) and generic SSRIs (61). In keeping with the associated restrictions of this analytical approach, all used a short time horizon, typically 6 months or less. The decision trees largely followed a similar structure, modeling three possible outcomes: remission, response (with no remission), and non-response. A representation of the generic decision tree structure is shown in Figure 2.

**TABLE 3A:** Model characteristics for economic evaluations of non-pharmacological neuromodulation.

References	Comparators	Study design	Model type	Horizon	Main health states modeled
Nguyen and Gordon (62)	rTMS TAU (pharmacotherapy)	CUA	Markov microsimulation	36 months	Acute treatment Full remission Partial remission No response/relapse Post treatment ECT Post-treatment lithium augmentation Acute episode hospitalization Death
Simpson et al. (66)	TMS Pharmacotherapy Sham TMS	CUA	Markov model	12 months	Well: MADRS 0-9 Mild: MADRS 10-17 Moderate: MADRS 18-27 Severe: MADRS > 28
Ross et al. (64)	ECT at different lines of therapy TAU (pharmacotherapy)	CUA	Markov model	48 months	Remission Response Relapse Non-response
Fitzgibbon et al. (56)	rTMS ECT combined rTMS + ECT stepped pathway	CUA	Markov microsimulation	Lifetime	Acute treatment Remission Maintenance treatment Severe depression Death
Ghiasvand et al. (57)	rTMS ECT	CEA CUA	Decision tree	7 months	Remission Relapse
Health Quality Ontario (58)	rTMS ECT sham rTMS	CUA	Decision tree	6 months	Non-response Response Remission
Unit University of Calgary (59)	rTMS ECT	CUA	Decision tree	6 weeks	Response Remission Relapse
Kozel et al. (60)	rTMS ECT combined rTMS + ECT stepped pathway	CUA	Decision tree	12 months	Non-response Response Continued response Relapse
Vallejo-Torres et al. (67)	ECT rTMS combined rTMS + ECT stepped pathway	CUA	Markov model	12 months	Acute treatment/relapse Continuation treatment Stable with/without treatment Moderate depression Severe depression Death
Xie et al. (69)	rTMS ECT sham rTMS + TAU	CUA	Decision tree	6 months	Non-response Response Remission
Galletly et al. (52)	rTMS ECT	CUA	Markov microsimulation	36 months	Acute treatment Full remission Partial remission No response/relapse Post treatment ECT Post-treatment lithium augmentation Acute episode hospitalization Death
Zhao et al. (71)	rTMS ECT	CUA	Markov model	12 months	Remission Non-remission Relapse Stable (remission) Severe depression Death (suicide or other causes)

**TABLE 3B:** Model characteristics for economic evaluations of pharmacological interventions.

References	Comparators	Study design	Model type	Horizon	Main health states modeled
Atlas et al. (53)	Esketamine + Antidepressant Antidepressant	CUA	Markov model	Lifetime	Non-response Partial response Response Remission Treatment failure Relapse Discontinuation Death
Desai et al. (54)	Esketamine + Antidepressant Placebo + Antidepressant	CEA	Markov model	12 months	Response Remission Relapse
Ross and Soeteman (65)	Esketamine + TAU TAU	CUA	Markov model	60 months	Remission Response Relapse Non-response
Edwards et al. (55)	AAP + Antidepressant Lithium + Antidepressant	CUA	Markov model	12 months	Non-response Response (continue/discontinue) Remission (continue/discontinue) Relapse
Malone (61)	Antidepressants (escitalopram, paroxetine CR, sertraline, venlafaxine) Generic SSRIs	CEA	Decision tree	6 months	Non-response Response Remission
Olgiati et al. (63)	Sequenced treatment (either switch or augment following citalopram non-response) Continued citalopram	CUA	Markov model	26 weeks	Acute depression/non-remission/relapse Remission No treatment
Wang et al. (68)	Hypothetical monotherapy SSRI + AAP	CUA	Markov model	12 months	Full remission discontinued Full remission Partial remission discontinued Partial remission In episode discontinued Relapse discontinued
Young et al. (70)	Vortioxetine SSRIs, SNRIs, agomelatine	CUA	Markov model	24 months	Non-response Response Remission Recovery Long-term AEs

There were several notable variations from this structure. Both Kozel et al. (60) and Ghiasvand et al. (57) assumed that any response equated to full remission. This is a significant limitation as it does not allow for partial improvements in symptoms, and thereby is likely to overestimate the benefits of interventions.

Kozel’s model also allowed for relapse. The model described by Malone et al. (61) compared the costs and consequences of various pharmaceutical treatment regimens, and augmented this generic structure with further steps that considered adverse events (AEs), and treatment changes. While four of the six evaluations described the conceptualization of the model structure, none described the rationale for selecting a decision tree approach, and only half described any structural assumptions or indicated that any validation assessment was undertaken.

**FIGURE 3:**
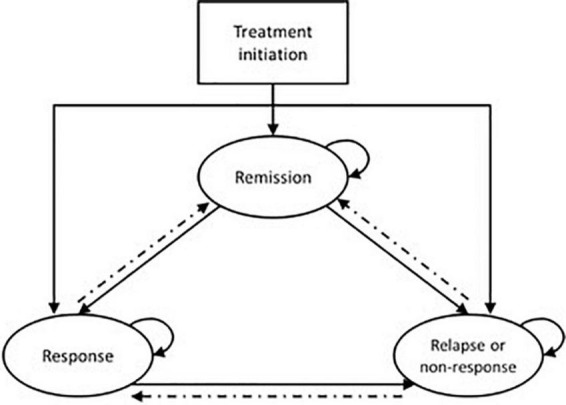
Generic Markov model structure for economic evaluations in TRD. Patients solid lines indicate pathways that are common amongst the majority of models; dashed lines indicate pathways that are included in a subset of models.

Twelve evaluations used Markov cohort models, and three extended this approach with more sophisticated Markov microsimulation models (all for neuromodulation interventions). A key characteristic of this extension is that it enables the tracking of individual patient characteristics or event history through the model. Most Markov models had a minimum horizon of 12 months, but only two had a lifetime horizon (53, 56). A similar “base” generic structure, shown in shown in Figure 3, was used across the majority of models, with three key “health states”: remission, response, and relapse (and/or non-response).

Several evaluations extend beyond this base structure, varying the levels of complexity and sophistication. Seven evaluations preceded the Markov model with a decision tree to represent a distinct acute phase of treatment. Other additional health states used (either as Markov health states or transition health states) included: death – particularly for models with a time horizon greater than 1 year (*n* = 7); treatment change (*n* = 7); severe depression (*n* = 6); discontinuation (*n* = 7); adverse events (*n* = 4); hospitalization (*n* = 2). Only one evaluation used an entirely different structure, modeling health states defined by four different levels of severity of depression (defined by MADRS score) (39).

The reporting of these models was generally good, with a majority describing a rationale for model structure (*n* = 9) and structural assumptions (*n* = 8). Nevertheless, some aspects of the health states included or omitted require some important limiting assumptions. Only seven models accounted for discontinuation of treatment, and none of those omitting discontinuation justified the omission. Discontinuation might feasibly be rolled into the “non-response” health state, however, this was not explicitly stated in any evaluation that omitted a “discontinued” health state; these may consequently overestimate treatment benefits by failing to account for discontinued patients. Of those evaluations that did include discontinuation, four either did not distinguish between discontinuation related to AEs or lack of efficacy, or assumed discontinuation due to AEs to be embedded in loss of treatment effect (53, 55, 67, 68). These four evaluations therefore considered AEs implicitly, but assumed no continued impact on quality of life beyond that of discontinuation due to lack of efficacy – an assumption that may not hold for severe or long-lasting AEs. AEs were considered explicitly in only five evaluations. Two considered both costs and utility decrements associated with AEs (52, 62), two considered only utility decrement (64), and one considered only costs (71). The majority did not model AEs and in most cases a rationale was not given, although it was suggested in two evaluations that the impact of AEs was expected to be limited, and similar between comparators (55, 59). While this assumption may be true of some comparators, it is an important structural assumption to validate, as omission will bias toward those interventions that have higher rates of AEs.

#### 4.7.1. Utility data

There was considerable heterogeneity used in approaches to sourcing utility data for use in cost utility models: 11 different sources, using six different methods of deriving utility (EQ-5D-3L [*n* = 13]; standard gamble [*n* = 4]; McSad [*n* = 2]; SF-6D [*n* = 1]; HUI-3 [*n* = 1]) were identified. The two main sources of utility values were studies by Sapin et al. (93) (*n* = 7) and Revicki et al. (92) (*n* = 4). The utility values derived from these two studies were based on MDD rather than TRD populations, and report only crude unadjusted values. Notably, only two evaluations used values derived from patients with TRD (53, 70). Use of values derived from the broader MDD population was driven by the scarcity of health-related quality of life data specific to patients with TRD. Ideally, values used should be population specific (94, 95) – the extent to which MDD values generalize to TRD is unknown. All evaluations that modeled the disutility of adverse events (*n* = 3) drew these values from a study of AEs associated with SSRIs in MDD (96). As a consequence of the heterogeneity in sources used (and concomitantly, the heterogeneity of the populations from which the source data were drawn), there was also considerable variability in the values used for common health states: baseline depression, 0.25–0.55; remission, 0.76–0.91; response 0.71–0.76; no response 0.52–0.58; relapse 0.30–0.63. Utility values used in the models, and their information sources are summarized in Supplementary Table 4.

### 4.8. Results of evaluations

Table 4 summarizes evaluation results. Consistency of results varied across interventions. Four evaluations comparing neuromodulation (rTMS or ECT) to TAU consistently found the intervention to be cost-effective, and a dominant strategy (both more effective and less costly) in three evaluations. Direct comparisons of ECT and rTMS, however, were less consistent: six favored ECT and four favored rTMS. The source of these variations is not immediately clear; however, those that favored ECT tended to have a shorter (<12 month) time horizon, which may not have been long enough to capture benefits of maintenance treatment with rTMS. Those that adopted a societal perspective tended to favor rTMS, reflecting the higher indirect costs (care, time off work) of ECT. There is no clear indication that study or model design biased results in either direction. Notably, over half of these evaluations did not explicitly define the patient population in terms of severity or number of previously failed treatments. There was variation in the treatment protocol used for rTMS, which is likely to have a considerable impact on costs, as will the extent to which capital costs are attributed across different evaluations.

**TABLE 4A:** Summary of authors’ conclusions, and key drivers of uncertainty for economic evaluations of non-pharmacological neuromodulation interventions.

References	Comparators	Evaluation conclusion	Key drivers of uncertainty^1^
Nguyen and Gordon (62) Australia	rTMS TAU (pharmacotherapy)	rTMS dominates (cheaper and more effective) TAU	Probability of response, remission & relapse
Simpson et al. (66) USA	TMS Pharmacotherapy Sham TMS	rTMS is likely to be cost-effective compared to sham rTMS rTMS dominates pharmacotherapy	Cost of rTMS
Ross et al. (64) USA	ECT at different lines of therapy TAU (pharmacotherapy)	Offering ECT after 2 failed lines of pharmacotherapy/psychotherapy likely to be the most cost-effective algorithm	**Cost of ECT cost** **Utility value for non-response** **Probability of response**
McDonald et al. (72) USA	ECT TAU	ECT dominates TAU	None identified
Fitzgibbon et al. (56) Canada	rTMS ECT combined rTMS + ECT stepped pathway	rTMS dominates ECT in first line	**Frequency of rTMS administration**
Ghiasvand et al. (57) Iran	rTMS ECT	ECT more cost effective than rTMS	Costs of interventions
Health Quality Ontario (58) Canada	rTMS ECT sham rTMS	ECT likely to be cost effective compared to rTMS	**Probability of response to ECT and rTMS**
Unit University of Calgary (59) Canada	rTMS ECT	rTMS dominates ECT	**Probability of response & remission** **rTMS treatment cost**
Kozel et al. (60) USA	rTMS ECT combined rTMS + ECT stepped pathway	ECT unlikely to be more cost effective than rTMS Stepped rTMS-ECT pathway dominates	Probability of response & remission Intervention costs
Vallejo-Torres et al. (67) Spain	ECT rTMS combined rTMS + ECT stepped pathway	ECT dominates rTMS Stepped pathway unlikely to be cost effective	None identified
Xie et al. (69) Canada	rTMS ECT sham rTMS + TAU	Low probability of rTMS being cost effective using a non-inferiority framework (and a 75% preservation of effectiveness threshold)	None identified
Galletly et al. (52) Australia	rTMS ECT	rTMS unlikely to be cost effective compared to ECT	**Probability remission after treatment** **Probability remission after hospitalization** **Number of rTMS and ECT sessions per treatment course**
Zhao et al. (71) Singapore	rTMS ECT	rTMS likely to be cost effective compared to ECT	**Probability remission after** **Cost for hospitalization due to ECT**
Knapp et al. (75) UK	rTMS ECT	Very low probability that rTMS is cost effective compared to ECT	None identified

^1^Items in bold denote studies that conducted a comprehensive one-way uncertainty analysis.

**TABLE 4B:** Summary of authors’ conclusions, and key drivers of uncertainty for economic evaluations of pharmacological interventions.

References	Comparators	Evaluation conclusion	Key drivers of uncertainty^2^
Atlas et al. (53) USA	Esketamine + Antidepressant Antidepressant	Esketamine unlikely to be cost effective	**Utilities for severe depression** **Probability of continued effect** **Probability of discontinuing therapy if effective**
Desai et al. (54) USA	Esketamine + Antidepressant Placebo + Antidepressant	Esketamine cost per remitter ($14-39k) is cost-effective	Probability of relapse free remission
Ross and Soeteman (65) USA	Esketamine + TAU TAU	Esketamine unlikely to be cost effective	**Probability of response and remission**
Edwards et al. (55) UK	AAP + Antidepressant Lithium + Antidepressant	Lithium dominates AAP (though subject to considerable uncertainty)	**Probability of acute efficacy** **Probability of discontinuation**
Malone (61) USA	Antidepressants (escitalopram, paroxetine CR, sertraline, venlafaxine) Generic SSRIs	Cost per remitter lowest for venlafaxine	None identified
Olgiati et al. (63) USA	Sequenced treatment (switch/augment following citalopram non-response) Continued citalopram	Sequenced treatment likely to be cost effective compared to remaining on citalopram	Utility values for acute depression and remitted depression
Wang et al. (68) UK	Hypothetical monotherapy SSRI + AAP	Hypothetical monotherapy dominates SSRI + AAP	**Probability of response and remission**
Young et al. (70) UK	Vortioxetine SSRIs, SNRIs, agomelatine	Vortioxetine in the third line likely to be cost effective compared to SSRIs	Secondary care costs
Kessler et al. (74) UK	mirtazapine placebo + TAU	No strong evidence that mirtazapine is cost-effective	None identified

^2^Items in bold denote studies that conducted a comprehensive one-way uncertainty analysis.

**TABLE 4C:** Summary of authors’ conclusions, and key drivers of uncertainty for economic evaluations of psychological interventions.

References	Comparators	Evaluation conclusion	Key drivers of uncertainty
Hollinghurst et al. (73) UK	CBT + TAU TAU	CBT + TAU is likely to be cost effective compared to TAU	QoL measure used (more cost-effective with EQ-5d-3L *cf.* SF-6D)
Wiles et al. (82) UK	CBT + TAU TAU	CBT + TAU is likely to be cost effective compared to TAU	None identified
Scott et al. (78) UK	CBT + TAU TAU	£12.50 per relapse-free day (conclusion depends on willingness to pay for a relapse free day)	None identified
Town et al. (81) Canada	ISTDP + TAU TAU	ISTDP likely to be cost effective compared to TAU	None identified
Shearer et al. (79) UK	RO-DBT + TAU TAU	Highly unlikely that RO-DBT is cost effective compared with TAU	None identified
Lynch et al. (76) UK	RO-DBT + TAU TAU	RO-DBT unlikely to be cost effective compared with TAU	None identified

**TABLE 4D:** Summary of authors’ conclusions, and key drivers of uncertainty for economic evaluations of service-level interventions.

References	Comparators	Evaluation conclusion	Key drivers of uncertainty
Morriss et al. (77) UK	Specialist depression service (SDS) TAU	SDS unlikely to be cost effective compared to TAU	None identified
Simon et al. (80) USA	Collaborative care program TAU	$21 per depression-free day – likely to be comparable ROI to other widely accepted medical interventions	None identified

Only three pharmacotherapy evaluations considered the same comparators (esketamine vs TAU). Two CUAs found that despite improved outcomes esketamine was unlikely to be cost effective. The third evaluation, which was industry-sponsored, used a CEA approach, and found esketamine was likely to be cost efficient. In addition to differences in analytical approaches used, the two CUAs had much longer time horizons (5 years and lifetime), compared to the 12 month CEA. It is likely that the consideration of relapse over those longer horizons had a significant impact on cost-effectiveness.

The evaluations evaluating psychological interventions, which were all trial-based, were generally consistent in their findings: two CUAs comparing CBT to TAU and one comparing ISTDP to TAU found that these interventions were likely to be cost effective; two CUAs comparing RO-DBT found that the intervention was highly unlikely to be cost effective. The key driver of the cost inefficiency for RO-DBT were the costs of intensive treatment.

We reviewed two trial-based evaluations of service-level interventions which are not directly comparable. A US-based collaborative care program was found to be cost effective (80), while an evaluation of a specialist depression service in the UK found limited additional benefits associated with the service and concluded it was unlikely to be cost-effective (77).

All except one evaluation explored uncertainty in parameters and/or results (72). Bootstrapping or similar methods were used to account for sampling uncertainty in almost all (*n* = 9) trial-based evaluations, while probabilistic sensitivity analyses were used to account for the joint uncertainty of all key parameters in over half (*n* = 13) of the model-based evaluations. Although most (*n* = 16) model-based evaluations conducted some degree of one-way sensitivity analysis, fewer than half (*n* = 9) conducted a comprehensive sensitivity analysis, incorporating all important variables. Key drivers of uncertainty included the probability of response and remission, utility values used for acute/severe depression, and cost of intervention (particularly for rTMS where number of treatment courses varied).

## 5. Discussion

The aim of this review was to appraise the literature systematically to describe the methods used in the economic evaluation of interventions for the management of TRD, to inform design and development of future evaluations in this field. We identified 31 evaluations, including 11 trial-based and 20 model-based evaluations. A broad range of interventions and designs were considered by the included evaluations, but almost half evaluated the cost effectiveness of neuromodulation interventions (rTMS and/or ECT), enhancing our ability to consider consistency of evaluation design, and the factors that most strongly influence results.

There was a distinct paucity of evidence relating to the economic evaluation of service-level interventions, with only two studies identified in the literature search. In their evaluation of a dedicated specialist depression service for TRD, Morris et al. (77) noted significant loss to follow-up during the trial and indicated the evaluation may have been underpowered to detect statistical improvements in symptoms at follow-up. It has been argued that the objective of economic evaluation is estimation of expected value of an intervention, and that decision making should therefore be based upon the weight of evidence, rather than the application of statistical inference rules (38, 97). Lack of statistical significance may, however, suggest that there is value in obtaining further evidence (97).

Despite a growing interest in the application of digital technologies in the management and delivery of mental health care (98), no economic evaluations of such interventions were identified. Recent studies suggest the implementation of digital technologies (e.g., virtual reality, artificial intelligence) may improve diagnosis, intervention delivery, monitoring, access to care, and potentially reduce costs (98, 99). Economic evidence supporting digital technologies in healthcare generally is underdeveloped: there is a clear need for early-stage economic evaluations to support the development of these promising approaches (100).

The quality of reporting as assessed by the CHEC criteria was generally good, and some aspects were found to be relatively consistent across the evaluations. Most evaluations considered comparable clinical outcomes – encompassing remission, response/non-response to treatment, and relapse. There was good agreement on the definitions and associated threshold for these outcomes, and these were assessed by a relatively small pool of clinical outcome measures. The resource criteria used to inform the estimation of direct costs including inpatient stays, outpatient appointments, and pharmaceutical costs, were reasonably uniform. Predominantly, however, there was a high level of heterogeneity in terms of evaluation design and sophistication, quality of evidence used (particularly with respect to health state utility data), time horizon, population considered, and cost perspective adopted. The impact of these inconsistencies is highlighted by the fact that despite the inclusion of 10 evaluations comparing rTMS and ECT, there is still inconclusive evidence as to the cost effectiveness of rTMS vs ECT.

Our findings are in general agreement with the literature relating to economic evaluation of MDD, where reviews have found the evidence for multiple interventions to be inconclusive due to inconsistencies in evaluation design and methodological quality (21, 22), and that the paucity of evidence related to long-term outcomes in TRD restricts our ability to inform the long-term value of interventions in TRD (23, 24). In order to inform future economic evaluations in TRD, and promote greater consistency among them, a number of linked methodological considerations are identified and good practices suggested.

### 5.1. Evaluation population and incorporation of patient heterogeneity

There was considerable variation in the definition used to describe the TRD population under study, with a fifth of evaluations providing no explicit definition. The absence of a standardized definition of the population reduces the validity of comparison and data synthesis across evaluations (101). However, one must acknowledge that the population is highly heterogeneous, in terms of both degree of treatment resistance, and medical and psychiatric co-morbid conditions (102). Evaluations that restrict the their population to a narrow definition or TRD, or that model a homogeneous cohort will limit generalizability of the findings. Despite this, very few model-based evaluations in this review explored the impact of patient heterogeneity – and where heterogeneity was considered, only a narrow range of aspects of heterogeneity were considered (age, gender, number of previous treatments). Equally, the under-reporting of severity at baseline is problematic when comparing economic evaluations, since this is likely to significantly impact outcomes (103).

To improve consistency across economic evaluations, we suggest that the widely used TRD definition of “failure to respond to two or more treatments at an adequate dose and duration” (9) be used as the base case for evaluation. Reflecting the concept that various degrees of resistance exist (102), more sophisticated evaluations might consider staging (for example by number of previous treatments), or at least characterizing the study population in this manner. Good practice guidelines for health economic models already highlight the importance of consideration of heterogeneity (47, 104). Cohort models can achieve this through sensitivity testing of results with alternative patient cohorts; more sophisticated patient-level models incorporate the facility to directly model heterogeneity.

### 5.2. Time horizon

The persistent and highly recurrent nature of TRD is not well reflected in many of the evaluations: the time horizon for most models was only 12 months, and the average for trials was 18 months. Only two evaluations used a lifetime horizon, extrapolating outcomes from clinical evaluations with follow-up periods of 12 months or less (53, 56). A key driver for the use of models in economic evaluation is to extrapolate the results of clinical trials to a longer-term horizon (47). In the context of TRD, a short time horizon may underestimate the cost effectiveness of an intervention by failing to account for smaller incremental improvements in mental health (accruing substantially with a longer horizon), or the improvements that persist beyond the evaluation horizon – for example, MDD patients receiving cognitive therapy have been found to exhibit reduced relapse rates for up to 6 years (78). Conversely, bearing in mind the highly recurrent nature of TRD over periods of up to 36 months (105, 106), cost effectiveness might be overestimated through censoring of relapse or recurrence events. Extrapolation implicitly introduces additional uncertainty into the model, but one must balance the impact of that additional uncertainty on results against the benefits of decision support that reflects the longer-term costs and consequences of the intervention in question.

### 5.3. Analytical framework

Most evaluations included in this study used a CUA design, typically estimating incremental QALY changes associated with each alternative, with only five (mostly older evaluations) using only a CEA or CCA design. While the CEA approach has advantages – the results can be more intuitive for decision makers, and uncertainty is reduced since conversion of outcome measures to utility scores is not required – the results are of lesser value than those of a CUA for informing resource allocation decisions. Firstly, there is no immediately obvious decision rule: at what threshold of cost should a depression-free day be considered cost effective, for example? Perhaps more important, though, is the facility enabled by CUA to evaluate the cost effectiveness of an intervention within the whole healthcare sector. Mental healthcare provision is underfunded globally (107), and budgets for provision of mental healthcare are typically not ringfenced, but must compete with other healthcare priorities. To justify support for novel interventions, commissioners must be able to appraise the value of those interventions within the context of these competing priorities – e.g., mental health vs cardiovascular disease.

### 5.4. Summary measures of benefit

The most common economic outcome measure was the QALY, in most cases estimated using the EQ-5D-3L measure. Model-based evaluations predominantly used low-quality evidence to inform this parameter: sources were typically outdated, used unsophisticated valuation methods, and were usually drawn from the broader MDD population, rather than TRD specific. There is good evidence that an increased number of treatment failures within an episode is associated with both increased depression severity and decreased HRQoL (8). This would indicate that HRQoL in TRD follows a somewhat distinct profile from the broader MDD population, and highlights the importance of using values specific to the population under study. Generic preference-based HRQoL measures are increasingly deployed in interventional evaluations (including eight described in this review): synthesis of contemporary data specific to the TRD population should therefore considered for future economic evaluations.

Generic measures are typically recommended over condition-specific measures, since they facilitate comparable outcome collection across the healthcare spectrum, and (due to their brevity) are easy to collect (93). Despite their widespread use, however, there is a growing consensus amongst health economists working in mental health that generic measures such as the EQ-5D are not sufficiently sensitive to capture important changes in symptoms, functioning, or wellbeing in mental health conditions (108). While there is evidence that these issues may be valid in depression, concordance between generic HRQoL measures and clinical measures has been shown to reduce with severity (109). Partly in response to these concerns, there has been increased focus on measurement of wellbeing and quality of life in mental health (110), but to date, there exists no mental health domain-specific preference-based measure that has been sufficiently validated that it can be recommended as an alternative to the EQ-5D or the SF-6D. In the absence of such a measure, the quality of the evidence used to inform EQ-5D generated utility data is of particular importance, and extensive sensitivity testing of utility values is imperative. It should be noted that increasingly, the updated EQ-5D-5L (rather than the -3L) measure is used in interventional studies, owing to is superior psychometric properties (111). The value of supplementing a CUA with a secondary CEA or CCA analysis (for example incorporating mental-health specific outcomes, or patient preferences), in order to increase confidence in results, may additionally be considered.

Where a CEA approach was adopted, various outcomes were used (cost per remitter, cost per depression-free day, cost per relapse prevented, or simply incremental change in outcome). Cost per remission is arguably a more intuitive measure to present to decision makers, and conversion of the cost per unit change to this measure should be relatively straightforward, providing adequate availability of information.

### 5.5. Patient preference and priorities

Recent years have seen increasing interest in the adoption of a “values-based” framework for delivery of mental health care, explicitly incorporating the preferences, priorities, and values of mental health service users (112, 113). The incorporation of patient preferences in decisions related to resource allocation is justifiable on grounds of both ethics (since patients have agency in the decisions that affect their health), and on improving outcomes (patients are more likely to engage with interventions that match their preferences) (114). Despite this, none of the evaluations described incorporated patient values, preferences, or priorities in the presentation of their analysis. The HTA report by Atlas et al. (53) incorporated feedback from patient advocates, importantly highlighting concerns that the clinical outcome measures typically used do not reflect the full burden of TRD, and calling for the incorporation of measures of impact on work, productivity, disability, and family or caregiver wellbeing. Elsewhere, patients have argued that remission is more accurately described by the presence of positive mental health features (optimism, vigor, and self-confidence) than the absence of symptoms (115). Although currently not a pre-requisite for HTA submissions, or best practice guidance, the growing recognition of the importance of the perspective of the patient in resource allocation decisions warrants serious consideration of how this might be incorporated explicitly in future economic evaluations. Longer-term objectives might consider the co-development of outcome measures that better reflect patient priorities; more immediately, methods such as discrete-choice experiments may be used to directly elicit and value both health and non-health impacts of interventions, facilitating direct incorporation of patient preferences in economic evaluations (116).

### 5.6. Reporting of resource use and cost data

Resource use in economic evaluation is highly context-specific – owing to the breadth of interventions, jurisdictions and cost perspectives considered by the evaluations in this study, a granular critical evaluation and comparison of resource use is unlikely to be informative. Focusing instead on broader resource item considerations, we found a reasonable level of consistency for direct costs across the evaluations. A third of the evaluations reviewed included indirect non-healthcare costs, although with considerable variation in the items included. In many cases this simply including productivity gains or losses which, when measured over relatively short time horizons, had a relatively small impact on results compared to the healthcare perspective. A minority considered a more comprehensive set of indirect costs. Variability in indirect costs that contribute to the broader “societal” perspectives is in part a reflection of the different contexts in which these evaluations were conducted: out-of-pocket costs, reliance on informal care, or transport costs may vary significantly between jurisdictions and in some cases may be so negligible that they are not considered for inclusion.

Good practice guidance relating to selection of costs for inclusion in economic evaluations recommends that either all relevant costs should be included, or (for more pragmatic studies) those costs that are most likely to meaningfully differ between comparators and thereby impact the result of the evaluation (47).

### 5.7. Perspective

The choice of cost perspective should be informed by the intended audience of the economic evaluation (47). Most commonly, the audience for economic evaluations is the payer; in the UK, NICE (whose remit is to determine if interventions should be funded by the NHS), requires that the perspective for economic evaluations should be that of the health service (104). Effective management of depression though, has been shown to have significantly greater impacts on productivity costs alone than on health care costs (21). When considering the global costs to society of poor mental health, choosing a narrow perspective that disregards those costs (or benefits) may be problematic, or even misleading.

Since mental health care is typically funded through public health care budgets, a health system perspective will be a pre-requisite for most decision makers, but we would reiterate the call from Knapp and Wong (21) that by providing a societal perspective in parallel, the broader societal impacts can also be taken into account. This broader perspective, however, is somewhat juxtaposed with our earlier recommendation that the primary analysis should use a CUA design. An immediate approach therefore might consider a secondary CCA analysis, adopting a societal perspective and reporting the non-health costs and benefits of alternatives.

### 5.8. Conceptualization and validation of model-based evaluations

None of the evaluations reviewed explicitly reported a formal conceptualization process, few presented a rationale for choice of model or model structure, and very few reported any robust validation of the model. The key health states described in most of the evaluations were consistent with established treatment goals of trials in MDD/TRD, including response, remission, and relapse (117). Sensitivity analyses of model-based evaluations frequently showed that it was these outcome parameters that were most likely to affect the results of the evaluations. Beyond these key endpoints, there was considerable variation in the structural complexity of model-based evaluations. Adverse events were rarely considered explicitly, although a minority of evaluations indicated that they had been considered and dismissed as having a negligible impact. Similarly, discontinuation was rarely considered, and where it was the reasons for discontinuation were poorly described.

Good practice guidance recommends an explicit process of conceptual modeling prior to implementation, to arrive at an appropriate scope for perspective, time horizon, choice of model type and structure, and which outcomes and costs to consider (118). The requirement to explicitly detail model conceptualization in reports has recently been added to the NICE HTA manual section 4.6.3 (104).

### 5.9. Limitations

This review restricted search criteria to English language only evaluations; by excluding foreign language records, our review may have limited consideration of aspects of economic evaluation that are prioritized differently in non-English speaking jurisdictions.

The review was deliberately designed with a “broad-brush” approach. Our aim was to develop a resource to inform the design of future economic evaluations in TRD agnostic of intervention, setting, or perspective. The review consequently incorporated all intervention types and all study design types; however, this introduces heterogeneity into the review, and limits the detail with which differences between evaluations may be explored. In keeping with the broad-brush approach, evaluation appraisal and recommendations are necessarily made at a generic level, and are not specific to context. Comparative evaluation of the results of included studies was conducted at a superficial level to illustrate how different evaluation design considerations may influence study conclusions. Where comparison of results is undertaken to inform resource allocation decisions, it is critical that context is accounted for. Key factors that should be considered in further detail in such comparisons include severity; number of previously failed treatments; treatment setting; and jurisdictional variations in resource costs and cost-effectiveness thresholds.

## 6. Conclusion

Consistent with reviews of economic evaluations in MDD (23), our review found that the economic evidence for interventions in TRD is underdeveloped, particularly so for service-level interventions. Where evidence does exist, it is hampered by inconsistency in study design, methodological quality, and availability of high quality long-term outcomes evidence. Consequently there is limited data available to reassure policy makers involved in commissioning interventions and services in TRD of their cost effectiveness.

To strengthen the evidence base, this review identifies a number of key considerations and challenges for the design of future economic evaluations. While some considerations may be addressed immediately (e.g., appropriately defining the evaluation population, and selection of appropriate time-horizon and perspective), we also identify longer term challenges related to methodology development and building consensus in the research community to promote consistency in study design. The lack of long-term outcomes data limits the value of current economic evaluations. In particular we identified a need for more robust health-state utility data specific to TRD; consensus for a core outcome set that incorporates the measures from which these are derived would be a significant step forward.

Reflecting the growing recognition of the importance of incorporating the values of the patient in resource allocation decisions, we also suggest there is a need to develop methods to incorporate those values in economic evaluation frameworks systematically.

## Data availability statement

The original contributions presented in this study are included in this article/Supplementary material; further inquiries can be directed to the corresponding author.

## Author contributions

LJ and CW designed the research programme this review belongs to. LH, JP, and RAC developed the protocol and study design. LH and RAC conducted the literature search, screening of reports, data extraction, analysis, and drafted the manuscript. RNC contributed to the interpretation of literature review and critical revision of the manuscript. All authors reviewed and contributed to the final draft of this manuscript.

## Footnotes

1
https://www.rayyan.ai/

## References

[1] World Health Organization. *Depression Factsheet.* Geneva: World Health Organization (2021).

[2] Proudman D Greenberg P Nellesen D. The growing burden of major depressive disorders (MDD): implications for researchers and policy makers. *Pharmacoeconomics.* (2021) 39:619–25. 10.1007/s40273-021-01040-7 34013439 PMC8134814

[3] Santomauro D Herrera A Shadid J Zheng P Ashbaugh C Pigott D Global prevalence and burden of depressive and anxiety disorders in 204 countries and territories in 2020 due to the COVID-19 pandemic. *Lancet.* (2021) 398:1700–12. 10.1016/S0140-6736(21)02143-7 34634250 PMC8500697

[4] Esterwood E Saeed S. Past epidemics, natural disasters, COVID19, and mental health: learning from history as we deal with the present and prepare for the future. *Psychiatr Q.* (2020) 91:1121–33. 10.1007/s11126-020-09808-4 32803472 PMC7429118

[5] Kathirvel N. Post COVID-19 pandemic mental health challenges. *Asian J Psychiatr.* (2020) 53:102430. 10.1016/j.ajp.2020.102430 33264840 PMC7507979

[6] Trivedi M Fava M Wisniewski S Thase M Quitkin F Warden D Medication augmentation after the failure of SSRIs for depression. *N Engl J Med.* (2006) 354:1243–52. 10.1056/NEJMoa052964 16554526

[7] Rush A Trivedi M Wisniewski S Nierenberg A Stewart J Warden D Acute and longer-term outcomes in depressed outpatients requiring one or several treatment steps: a STAR*D report. *Am J Psychiatry.* (2006) 163:1905–17. 10.1176/ajp.2006.163.11.1905 17074942

[8] Johnston K Powell L Anderson I Szabo S Cline S. The burden of treatment-resistant depression: a systematic review of the economic and quality of life literature. *J Affect Disord.* (2019) 242:195–210. 10.1016/j.jad.2018.06.045 30195173

[9] Gaynes B Lux L Gartlehner G Asher G Forman-Hoffman V Green J Defining treatment-resistant depression. *Depress Anxiety.* (2020) 37:134–45. 10.1002/da.22968 31638723

[10] Demyttenaere K Van Duppen Z. The impact of (the Concept of) treatment-resistant depression: an opinion review. *Int J Neuropsychopharmacol.* (2019) 22:85–92. 10.1093/ijnp/pyy052 29961822 PMC6368367

[11] Akil H Gordon J Hen R Javitch J Mayberg H McEwen B Treatment resistant depression: a multi-scale, systems biology approach. *Neurosci Biobehav Rev.* (2018) 84:272–88. 10.1016/j.neubiorev.2017.08.019 28859997 PMC5729118

[12] Bower P Gilbody S. Stepped care in psychological therapies: access, effectiveness and efficiency: narrative literature review. *Br J Psychiatry.* (2005) 186:11–7. 10.1192/bjp.186.1.11 15630118

[13] National Institute for Health and Care Excellence. *Depression in Adults: Treatment and Management.* London: National Institute for Health and Care Excellence (2022). 35977056

[14] McIntyre R Millson B Power G. Burden of treatment resistant depression (TRD) in patients with major depressive disorder in Ontario using institute for clinical evaluative sciences (ICES) databases: economic burden and healthcare resource utilization. *J Affect Disord.* (2020) 277:30–8. 10.1016/j.jad.2020.07.045 32791390

[15] Jaffe D Rive B Denee T. The humanistic and economic burden of treatment-resistant depression in Europe: a cross-sectional study. *BMC Psychiatry.* (2019) 19:247. 10.1186/s12888-019-2222-4 31391065 PMC6686569

[16] Fekadu A Wooderson S Markopoulo K Donaldson C Papadopoulos A Cleare A. What happens to patients with treatment-resistant depression? A systematic review of medium to long term outcome studies. *J Affect Disord.* (2009) 116:4–11. 10.1016/j.jad.2008.10.014 19007996

[17] Olchanski N McInnis Myers M Halseth M Cyr P Bockstedt L Goss T The economic burden of treatment-resistant depression. *Clin Ther.* (2013) 35:512–22. 10.1016/j.clinthera.2012.09.001 23490291

[18] Sussman M O’Sullivan AK Shah A Olfson M Menzin J. Economic burden of treatment-resistant depression on the U.S. health care system. *J Manag Care Spec Pharm.* (2019) 25:823–35. 10.18553/jmcp.2019.25.7.823 31232205 PMC10398213

[19] Mrazek D Hornberger J Altar C Degtiar I. A review of the clinical, economic, and societal burden of treatment-resistant depression: 1996-2013. *Psychiatr Serv.* (2014) 65:977–87. 10.1176/appi.ps.201300059 24789696

[20] Perez-Sola V Roca M Alonso J Gabilondo A Hernando T Sicras-Mainar A Economic impact of treatment-resistant depression: a retrospective observational study. *J Affect Disord.* (2021) 295:578–86. 10.1016/j.jad.2021.08.036 34509073

[21] Knapp M Wong G. Economics and mental health: the current scenario. *World Psychiatry.* (2020) 19:3–14. 10.1002/wps.20692 31922693 PMC6953559

[22] Grochtdreis T Brettschneider C Wegener A Watzke B Riedel-Heller S Harter M Cost-effectiveness of collaborative care for the treatment of depressive disorders in primary care: a systematic review. *PLoS One.* (2015) 10:e0123078. 10.1371/journal.pone.0123078 25993034 PMC4437997

[23] Zimovetz E Wolowacz S Classi P Birt J. Methodologies used in cost-effectiveness models for evaluating treatments in major depressive disorder: a systematic review. *Cost Eff Resour Alloc.* (2012) 10:1. 10.1186/1478-7547-10-1 22296830 PMC3293043

[24] Altunkaya J Lee J Tsiachristas A Waite F Freeman D Leal J. Appraisal of patient-level health economic models of severe mental illness: systematic review. *Br J Psychiatry.* (2022) 220:2. 10.1192/bjp.2021.121 35049466 PMC7612275

[25] York Health Economics Consortium. *Economic Evaluation [online] York.* Heslington: York Health Economics Consortium (2016).

[26] York Health Economics Consortium. *Incremental Cost-Effectiveness Ratio (ICER) [online] York.* Heslington: York Health Economics Consortium (2016).

[27] Drummond M Sculpher M Claxton K Stoddart G Torrance G. *Introduction to Economic Evaluation. Methods for the Economic Evaluation of Health Care Programmes.* Oxford: Oxford University Press (2015).

[28] York Health Economics Consortium. *Utility [online].* Heslington: York Health Economics Consortium (2016).

[29] Brazier J Ratcliffe J Saloman J Tsuchiya A. *Introduction to the Measurement and Valuation of Health. Measuring and Valuing Health Benefits for Economic Evaluation.* Oxford: Oxford University Press (2017). 10.1093/med/9780198725923.001.0001

[30] Drummond M Sculpher M Claxton K Stoddart G Torrance G. *Making Decisions in Health Care. Methods for the Economic Evaluation of Health Care Programmes.* Oxford: Oxford University Press (2015).

[31] Sanders G Neumann P Basu A Brock D Feeny D Krahn M Recommendations for conduct, methodological practices, and reporting of cost-effectiveness analyses: second panel on cost-effectiveness in health and medicine. *JAMA.* (2016) 316:1093–103. 10.1001/jama.2016.12195 27623463

[32] Culyer A Chalkidou K Teerawattananon Y Santatiwongchai B. Rival perspectives in health technology assessment and other economic evaluations for investing in global and national health. Who decides? Who pays? *F1000Res.* (2018) 7:72. 10.12688/f1000research.13284.1 29904588 PMC5961761

[33] Ramsey S Willke R Glick H Reed S Augustovski F Jonsson B Cost-effectiveness analysis alongside clinical trials II—an ISPOR good research practices task force report. *Value Health.* (2015) 18:161–72. 10.1016/j.jval.2015.02.001 25773551

[34] Briggs A Sculpher M Claxton K. *Decision Modelling for Health Economic Evaluation.* Oxford: Oup Oxford (2006).

[35] York Health Economics Consortium. *Time Horizon [online] York.* Heslington: York Health Economics Consortium (2016).

[36] Drummond M Sculpher M Claxton K Stoddart G Torrance G. *Using Clinical Studies as Vehicles for Economic Evaluation. Methods for the Economic Evaluation of Health Care Programmes.* Oxford: Oxford University Press (2015).

[37] Petrou S Gray A. Economic evaluation alongside randomised controlled trials: design, conduct, analysis, and reporting. *BMJ.* (2011) 342:d1548. 10.1136/bmj.d1548 21474510 PMC3230107

[38] Briggs A. Economic evaluation and clinical trials: size matters. *BMJ.* (2000) 321:1362. 10.1136/bmj.321.7273.1362 11099268 PMC1119102

[39] Fenwick E Byford S. A guide to cost-effectiveness acceptability curves. *Br J Psychiatry.* (2005) 187:106–8. 10.1192/bjp.187.2.106 16055820

[40] Michalowsky B Hoffmann W Kennedy K Xie F. Is the whole larger than the sum of its parts? Impact of missing data imputation in economic evaluation conducted alongside randomized controlled trials. *Eur J Health Econ.* (2020) 21:717–28. 10.1007/s10198-020-01166-z 32108274 PMC7366573

[41] Jalali A Tamimi R McPherson S Murphy S. Econometric issues in prospective economic evaluations alongside clinical trials: combining the nonparametric bootstrap with methods that address missing data. *Epidemiol Rev.* (2022) 44:67–77. 10.1093/epirev/mxac006 36104860 PMC10362933

[42] Brand J van Buuren S le Cessie S van den Hout W. Combining multiple imputation and bootstrap in the analysis of cost-effectiveness trial data. *Stat Med.* (2019) 38:210–20. 10.1002/sim.7956 30207407 PMC6585698

[43] Drummond M Sculpher M Claxton K Stoddart G Torrance G. *Economic Evaluation Using Decision-Analytic Modelling. Methods for the Economic Evaluation of Health Care Programmes.* Oxford: Oxford University Press (2015).

[44] York Health Economics Consortium. *Decision Tree [online].* Heslington: York Health Economics Consortium (2016).

[45] York Health Economics Consortium. *Markov Model [online].* Heslington: York Health Economics Consortium (2016).

[46] York Health Economics Consortium. *Micro-Simulation [online].* Heslington: York Health Economics Consortium (2016).

[47] Drummond M Sculpher M Claxton K Stoddart G Torrance G. *Methods for the Economic Evaluation of Health Care Programmes.* Oxford: Oxford University Press (2015).

[48] Page M McKenzie J Bossuyt P Boutron I Hoffmann T Mulrow C The PRISMA 2020 statement: an updated guideline for reporting systematic reviews. *BMJ.* (2021) 372:n71. 10.1136/bmj.n71 33782057 PMC8005924

[49] Evers S Goossens M de Vet H van Tulder M Ament A. Criteria list for assessment of methodological quality of economic evaluations: consensus on health economic criteria. *Int J Technol Assess Health Care.* (2005) 21:240–5. 10.1017/S0266462305050324 15921065

[50] van Mastrigt G Hiligsmann M Arts J Broos P Kleijnen J Evers S How to prepare a systematic review of economic evaluations for informing evidence-based healthcare decisions: a five-step approach (part 1/3). *Expert Rev Pharmacoecon Outcomes Res.* (2016) 16:689–704. 10.1080/14737167.2016.1246960 27805469

[51] Philips Z Ginnelly L Sculpher M Claxton K Golder S Riemsma R Review of guidelines for good practice in decision-analytic modelling in health technology assessment. *Health Technol Assess.* (2004) 8:ii–iv, ix–xi, 1–158. 10.3310/hta8360 15361314

[52] Galletly C Clarke P Fitzgerald P Gill S Loo C Lyndon B *Repetitive Transcranial Magnetic Stimulation (rTMS), MSAC Application 1196.* Canberra, ACT: Commonwealth of Australia (2013).

[53] Atlas S Agboola F Fazioli K Kumar V Adair E Rind D *Esketamine for the Treatment of Treatment-Resistant Depression: Effectiveness and Value. Final Evidence Report.* Boston, MA: Institute for Clinical and Economic Review (2019).

[54] Desai U Kirson N Guglielmo A Le H Spittle T Tseng-Tham J Cost-per-remitter with esketamine nasal spray versus standard of care for treatment-resistant depression. *J Comp Eff Res.* (2021) 10:393–407. 10.2217/cer-2020-0276 33565893

[55] Edwards S Hamilton V Nherera L Trevor N. Lithium or an atypical antipsychotic drug in the management of treatment-resistant depression: a systematic review and economic evaluation. *Health Technol Assess.* (2013) 17:1–190. 10.3310/hta17540 24284258 PMC4781298

[56] Fitzgibbon K Plett D Chan B Hancock-Howard R Coyte P Blumberger D. Cost-utility analysis of electroconvulsive therapy and repetitive transcranial magnetic stimulation for treatment-resistant depression in Ontario. *Can J Psychiatry.* (2020) 65:164–73. 10.1177/0706743719890167 31801363 PMC7019468

[57] Ghiasvand H Moradi-Joo M Abolhassani N Ravaghi H Raygani S Mohabbat-Bahar S. Economic evaluation of resistant major depressive disorder treatment in Iranian population: a comparison between repetitive transcranial magnetic stimulation with electroconvulsive. *Med J Islam Repub Iran.* (2016) 30:330. 27390700 PMC4898845

[58] Health Quality Ontario. Repetitive transcranial magnetic stimulation for treatment-resistant depression: an economic analysis. *Ont Health Technol Assess Ser.* (2016) 16:1–51. PMC4808718 27110317

[59] Unit University of Calgary. *Repetitive Transcranial Magnetic Stimulation for Treatment-resistant DEPRESSION.* Calgary, AB: University of Calgary (2014). p. 2014

[60] Kozel F George M Simpson K. Decision analysis of the cost-effectiveness of repetitive transcranial magnetic stimulation versus electroconvulsive therapy for treatment of nonpsychotic severe depression. *CNS Spectr.* (2004) 9:476–82. 15162090

[61] Malone D. A budget-impact and cost-effectiveness model for second-line treatment of major depression. *J Manag Care Pharm.* (2007) 13:S8–18. 10.18553/jmcp.2007.13.s6-a.8 17874482 PMC10437458

[62] Nguyen K Gordon L. Cost-effectiveness of repetitive transcranial magnetic stimulation versus antidepressant therapy for treatment-resistant depression. *Value Health.* (2015) 18:597–604. 10.1016/j.jval.2015.04.004 26297087

[63] Olgiati P Bajo E Bigelli M Montgomery S Serretti A C.E.A.P (Cost-Effectiveness Analysis on Psychiatric Disorders)-Group. Challenging sequential approach to treatment resistant depression: cost-utility analysis based on the sequenced treatment alternatives to relieve depression (STAR()D) trial. *Eur Neuropsychopharmacol.* (2013) 23:1739–46. 10.1016/j.euroneuro.2013.08.008 24075716

[64] Ross E Zivin K Maixner D. Cost-effectiveness of electroconvulsive therapy VS pharmacotherapy/psychotherapy for treatment-resistant depression in the united states. *JAMA Psychiatry.* (2018) 75:713–22. 10.1001/jamapsychiatry.2018.0768 29800956 PMC6145669

[65] Ross E Soeteman D. Cost-effectiveness of esketamine nasal spray for patients with treatment-resistant depression in the United States. *Psychiatr Serv.* (2020) 71:988–97. 10.1176/appi.ps.201900625 32631129 PMC7920520

[66] Simpson K Welch M Kozel F Demitrack M Nahas Z. Cost-effectiveness of transcranial magnetic stimulation in the treatment of major depression: a health economics analysis. *Adv Ther.* (2009) 26:346–68. 10.1007/s12325-009-0013-x 19330495

[67] Vallejo-Torres L Castilla I Gonzalez N Hunter R Serrano-Perez P Perestelo-Perez L. Cost-effectiveness of electroconvulsive therapy compared to repetitive transcranial magnetic stimulation for treatment-resistant severe depression: a decision model. *Psychol Med.* (2015) 45:1459–70. 10.1017/S0033291714002554 25354790 PMC4413854

[68] Wang S Anderson I Mitchell D Johnson S Shiozawa A. Cost-effectiveness model for a hypothetical monotherapy vs standard of care in adult patients with treatment-resistant depression. *Clinicoecon Outcomes Res.* (2019) 11:257–70. 10.2147/CEOR.S181718 30936731 PMC6421973

[69] Xie X Falk L Brophy J Tu H Guo J Gajic-Veljanoski O A non-inferiority framework for cost-effectiveness analysis. *Int J Technol Assess Health Care.* (2019) 35:291–7. 10.1017/S0266462319000576 31337452

[70] Young A Evitt L Brignone M Diamand F Atsou K Campbell R Cost-utility evaluation of vortioxetine in patients with major depressive disorder experiencing inadequate response to alternative antidepressants in the United Kingdom. *J Affect Disord.* (2017) 218:291–8. 10.1016/j.jad.2017.04.019 28478358

[71] Zhao Y Tor P Khoo A Teng M Lim B Mok Y. Cost-effectiveness modeling of repetitive transcranial magnetic stimulation compared to electroconvulsive therapy for treatment-resistant depression in Singapore. *Neuromodulation.* (2018) 21:376–82. 10.1111/ner.12723 29143405

[72] McDonald W Phillips V Figiel G Marsteller F Simpson C Bailey M. Cost-effective maintenance treatment of resistant geriatric depression. *Psychiatr Ann.* (1998) 28:47–52. 10.3928/0048-5713-19980101-11

[73] Hollinghurst S Carroll F Abel A Campbell J Garland A Jerrom B Cost-effectiveness of cognitive-behavioural therapy as an adjunct to pharmacotherapy for treatment-resistant depression in primary care: economic evaluation of the CoBalT Trial. *Br J Psychiatry.* (2014) 204:69–76. 10.1192/bjp.bp.112.125286 24262818

[74] Kessler D Burns A Tallon D Lewis G MacNeill S Round J Combining mirtazapine with SSRIs or SNRIs for treatment-resistant depression: the MIR RCT. *Health Technol Assess.* (2018) 22:1–136. 10.3310/hta22630 30468145 PMC6287172

[75] Knapp M Romeo R Mogg A Eranti S Pluck G Purvis R Cost-effectiveness of transcranial magnetic stimulation vs. electroconvulsive therapy for severe depression: a multi-centre randomised controlled trial. *J Affect Disord.* (2008) 109:273–85. 10.1016/j.jad.2008.01.001 18262655

[76] Lynch T Hempel R Whalley B Byford S Chamba R Clarke P *Radically Open Dialectical Behaviour Therapy for Refractory Depression: The RefraMED RCT.* Southampton: NIHR Journals Library (2018). p. 12 10.3310/eme05070 30561967

[77] Morriss R Garland A Nixon N Guo B James M Kaylor-Hughes C Efficacy and cost-effectiveness of a specialist depression service versus usual specialist mental health care to manage persistent depression: a randomised controlled trial. *Lancet Psychiatry.* (2016) 3:821–31. 10.1016/S2215-0366(16)30143-2 27498098

[78] Scott J Palmer S Paykel E Teasdale J Hayhurst H. Use of cognitive therapy for relapse prevention in chronic depression. Cost-effectiveness study. *Br J Psychiatry.* (2003) 182:221–7. 10.1192/bjp.182.3.221 12611785

[79] Shearer J Lynch T Chamba R Clarke S Hempel R Kingdon D Refractory depression–cost-effectiveness of radically open dialectical behaviour therapy: findings of economic evaluation of RefraMED trial. *BJPsych Open.* (2019) 5:e64. 10.1192/bjo.2019.57 31352916 PMC6669879

[80] Simon G Katon W VonKorff M Unutzer J Lin E Walker E Cost-effectiveness of a collaborative care program for primary care patients with persistent depression. *Am J Psychiatry.* (2001) 158:1638–44. 10.1176/appi.ajp.158.10.1638 11578996

[81] Town J Abbass A Stride C Nunes A Bernier D Berrigan P. Efficacy and cost-effectiveness of intensive short-term dynamic psychotherapy for treatment resistant depression: 18-month follow-up of the Halifax depression trial. *J Affect Disord.* (2020) 273:194–202. 10.1016/j.jad.2020.04.035 32421603

[82] Wiles N Thomas L Turner N Garfield K Kounali D Campbell J Long-term effectiveness and cost-effectiveness of cognitive behavioural therapy as an adjunct to pharmacotherapy for treatment-resistant depression in primary care: follow-up of the CoBalT randomised controlled trial. *Lancet Psychiatry.* (2016) 3:137–44. 10.1016/S2215-0366(15)00495-2 26777773

[83] Brown S Rittenbach K Cheung S McKean G MacMaster F Clement F. Current and common definitions of treatment-resistant depression: findings from a systematic review and qualitative interviews. *Can J Psychiatry.* (2019) 64:380–7. 10.1177/0706743719828965 30763119 PMC6591751

[84] Zimmerman M Posternak M Chelminski I. Is the cutoff to define remission on the Hamilton rating scale for depression too high? *J Nerv Ment Dis.* (2005) 193:170–5. 10.1097/01.nmd.0000154840.63529.5d 15729106

[85] Hawley C Gale T Sivakumaran T. Hertfordshire neuroscience research g. defining remission by cut off score on the MADRS: selecting the optimal value. *J Affect Disord.* (2002) 72:177–84. 10.1016/S0165-0327(01)00451-7 12200208

[86] Zimmerman M Posternak M Chelminski I. Defining remission on the montgomery-Asberg depression rating scale. *J Clin Psychiatry.* (2004) 65:163–8. 10.4088/JCP.v65n0204 15003068

[87] Kennedy-Martin M Slaap B Herdman M van Reenen M Kennedy-Martin T Greiner W Which multi-attribute utility instruments are recommended for use in cost-utility analysis? A review of national health technology assessment (HTA) guidelines. *Eur J Health Econ.* (2020) 21:1245–57. 10.1007/s10198-020-01195-8 32514643 PMC7561556

[88] Janssen M Pickard A Golicki D Gudex C Niewada M Scalone L Measurement properties of the EQ-5D-5L compared to the EQ-5D-3L across eight patient groups: a multi-country study. *Qual Life Res.* (2013) 22:1717–27. 10.1007/s11136-012-0322-4 23184421 PMC3764313

[89] McLoughlin D Mogg A Eranti S Pluck G Purvis R Edwards D The clinical effectiveness and cost of repetitive transcranial magnetic stimulation versus electroconvulsive therapy in severe depression: a multicentre pragmatic randomised controlled trial and economic analysis. *Health Technol Assess.* (2007) 11:1–54. 10.3310/hta11240 17580003

[90] Bennett K Torrance G Boyle M Guscott R. Cost-utility analysis in depression: the McSad utility measure for depression health states. *Psychiatr Serv.* (2000) 51:1171–6. 10.1176/appi.ps.51.9.1171 10970923

[91] Feeny D Furlong W Torrance G Goldsmith C Zhu Z DePauw S Multiattribute and single-attribute utility functions for the health utilities index mark 3 system. *Med Care.* (2002) 40:113–28. 10.1097/00005650-200202000-00006 11802084

[92] Revicki D Wood M. Patient-assigned health state utilities for depression-related outcomes: differences by depression severity and antidepressant medications. *J Affect Disord.* (1998) 48:25–36. 10.1016/S0165-0327(97)00117-1 9495599

[93] Sapin C Fantino B Nowicki M Kind P. Usefulness of EQ-5D in assessing health status in primary care patients with major depressive disorder. *Health Qual Life Outcomes.* (2004) 2:20. 10.1186/1477-7525-2-20 15128456 PMC416488

[94] Brazier J Ratcliffe J Saloman J Tsuchiya A. *Design and Analysis of Health State Valuation Data for Model-Based Economic Evaluations and for Economic Evaluations Alongside Clinical Trials. Measuring and Valuing Health Benefits for Economic Evaluation.* Oxford: Oxford University Press (2017). 10.1093/med/9780198725923.003.0009

[95] Drummond M Sculpher M Claxton K Stoddart G Torrance G. *Identifying, Synthesizing, and Analysing Evidence for Economic Evaluation. Methods for the Economic Evaluation of Health Care Programmes.* Oxford: Oxford University Press (2015).

[96] Sullivan P Valuck R Saseen J MacFall H. A comparison of the direct costs and cost effectiveness of serotonin reuptake inhibitors and associated adverse drug reactions. *CNS Drugs.* (2004) 18:911–32. 10.2165/00023210-200418130-00006 15521793

[97] Claxton K. The irrelevance of inference: a decision-making approach to the stochastic evaluation of health care technologies. *J Health Econ.* (1999) 18:341–64. 10.1016/S0167-6296(98)00039-3 10537899

[98] Sin J Galeazzi G McGregor E Collom J Taylor A Barrett B Digital interventions for screening and treating common mental disorders or symptoms of common mental illness in adults: systematic review and meta-analysis. *J Med Internet Res.* (2020) 22:e20581. 10.2196/20581 32876577 PMC7495259

[99] Fulmer R Joerin A Gentile B Lakerink L Rauws M. Using psychological artificial intelligence (Tess) to relieve symptoms of depression and anxiety: randomized controlled trial. *JMIR Ment Health.* (2018) 5:e64. 10.2196/mental.9782 30545815 PMC6315222

[100] Milne-Ives M de Cock C Lim E Shehadeh M de Pennington N Mole G The effectiveness of artificial intelligence conversational agents in health care: systematic review. *J Med Internet Res.* (2020) 22:e20346. 10.2196/20346 33090118 PMC7644372

[101] Voineskos D Daskalakis Z Blumberger D. Management of treatment-resistant depression: challenges and strategies. *Neuropsychiatr Dis Treat.* (2020) 16:221–34. 10.2147/NDT.S198774 32021216 PMC6982454

[102] Fava M. Diagnosis and definition of treatment-resistant depression. *Biol Psychiatry.* (2003) 53:649–59. 10.1016/S0006-3223(03)00231-2 12706951

[103] Russell J Hawkins K Ozminkowski R Orsini L Crown W Kennedy S The cost consequences of treatment-resistant depression. *J Clin Psychiatry.* (2004) 65:341–7. 10.4088/JCP.v65n0309 15096073

[104] National Institute for Health and Care Excellence. *NICE Health Technology Evaluations: The Manual.* London: National Institute for Health and Care Excellence (2022).

[105] Gillain B Degraeve G Dreesen T De Bruecker G Buntinx E Beke D Real-world treatment patterns, outcomes, resource utilization and costs in treatment-resistant major depressive disorder: PATTERN, a retrospective cohort study in Belgium. *Pharmacoecon Open.* (2022) 6:293–302. 10.1007/s41669-021-00306-2 34782984 PMC8864045

[106] Lex H Nevers S Jensen E Ginsburg Y Maixner D Mickey B. Long-term quality of life in treatment-resistant depression after electroconvulsive therapy. *J Affect Disord.* (2021) 291:135–9. 10.1016/j.jad.2021.05.012 34038830 PMC8628522

[107] World Health Organization. *WHO Report Highlights Global Shortfall in Investment in Mental Health.* Geneva: World Health Organization (2021).

[108] Knapp M Mangalore R. The trouble with QALYs. *Epidemiol Psichiatr Soc.* (2007) 16:289–93. 10.1017/S1121189X00002451 18333423

[109] Brazier J Connell J Papaioannou D Mukuria C Mulhern B Peasgood T A systematic review, psychometric analysis and qualitative assessment of generic preference-based measures of health in mental health populations and the estimation of mapping functions from widely used specific measures. *Health Technol Assess.* (2014) 18:vii–viii, xiii–xxv, 1–188. 10.3310/hta18340 24857402 PMC4781324

[110] van Krugten F Feskens K Busschbach J Hakkaart-van Roijen L Brouwer W. Instruments to assess quality of life in people with mental health problems: a systematic review and dimension analysis of generic, domain- and disease-specific instruments. *Health Qual Life Outcomes.* (2021) 19:249. 10.1186/s12955-021-01883-w 34727928 PMC8561965

[111] Buchholz I Janssen M Kohlmann T Feng Y. A systematic review of studies comparing the measurement properties of the three-level and five-level versions of the EQ-5D. *PharmacoEconomics.* (2018) 36:645–61. 10.1007/s40273-018-0642-5 29572719 PMC5954044

[112] Woodbridge-Dodd K. Values-based practice in mental health and psychiatry. *Curr Opin Psychiatry.* (2012) 25:508–12. 10.1097/YCO.0b013e328359051c 22992551

[113] Baggaley M. Value-based healthcare in mental health services. *BJPsych Adv.* (2020) 26:198–204. 10.1192/bja.2019.82

[114] Swift J Mullins R Penix E Roth K Trusty W. The importance of listening to patient preferences when making mental health care decisions. *World Psychiatry.* (2021) 20:316–7. 10.1002/wps.20912 34505382 PMC8429341

[115] Zimmerman M McGlinchey J Posternak M Friedman M Attiullah N Boerescu D. How should remission from depression be defined? The depressed patient’s perspective. *Am J Psychiatry.* (2006) 163:148–50. 10.1176/appi.ajp.163.1.148 16390903

[116] Wildman J Wildman J. Combining health and outcomes beyond health in complex evaluations of complex interventions: suggestions for economic evaluation. *Value Health.* (2019) 22:511–7. 10.1016/j.jval.2019.01.002 31104728

[117] Rush A Thase M Dube S. Research issues in the study of difficult-to-treat depression. *Biol Psychiatry.* (2003) 53:743–53. 10.1016/S0006-3223(03)00088-X 12706958

[118] Roberts M Russell L Paltiel A Chambers M McEwan P Krahn M. Conceptualizing a model: a report of the ISPOR-SMDM modeling good research practices task force-2. *Med Decis Making.* (2012) 32:678–89. 10.1177/0272989X12454941 22990083

