# Supplementary material for: Economic evaluation of interventions for treatment-resistant depression: A systematic review
Source: Front Psychiatry. 2023 Feb 16;14:1056210. doi: 10.3389/fpsyt.2023.1056210 (PMC9979220; doi:10.3389/fpsyt.2023.1056210)
Supplement: Supplementary file 1 [file Table_1.DOCX]

# Supplementary Tables

**Table S1:** Electronic database search strategies

| Electronic databases | Economic evaluation search concept | Treatment resistant depression search concept |
| --- | --- | --- |
| OVID MEDLINE  OVID EMBASE | (analyses, cost benefit.mp OR analyses, cost-benefit.mp OR analyses, cost-utility.mp OR analyses, marginal.mp OR analysis, cost benefit.mp OR analysis, cost-benefit.mp OR analysis, cost-effectiveness.mp OR analysis, cost-utility.mp OR analysis, marginal.mp OR "benefits and costs".mp OR cost benefit.mp OR cost benefit analyses.mp OR cost benefit analysis.mp OR cost benefit data.mp OR cost effectiveness.mp OR cost effectiveness analysis.mp OR cost utility analysis.mp OR cost-benefit analyses.mp OR cost-benefit analysis.mp OR cost-benefit data.mp OR cost-effectiveness analysis.mp OR cost-utility analyses.mp OR cost-utility analysis.mp OR "costs and benefits".mp OR data, cost-benefit.mp OR economic evaluation.mp OR economic evaluations.mp OR effectiveness, cost.mp OR evaluation, economic.mp OR evaluations, economic.mp OR marginal analyses.mp OR marginal analysis.mp OR cost analysis.mp OR cost analyses.mp OR cost-consequence.mp OR cost consequence.mp) | (persistent depression.mp. OR depression, refractory.mp OR depression, therapy-resistant.mp OR depression, treatment resistant.mp OR depressions, refractory.mp OR depressions, therapy-resistant.mp OR depressions, treatment resistant.mp OR depressive disorder, treatment resistant.mp OR depressive disorder, treatment-resistant.mp OR depressive disorders, treatment-resistant.mp OR disorder, treatment-resistant depressive.mp OR disorders, treatment-resistant depressive.mp OR refractory depression.mp OR refractory depressions.mp OR resistant depression, treatment.mp OR resistant depressions, treatment.mp OR therapy resistant depression.mp OR therapy-resistant depression.mp OR therapy-resistant depressions.mp OR treatment resistant depression.mp OR treatment resistant depressions.mp OR treatment-resistant depressive disorder.mp OR treatment-resistant depressive disorders.mp) |
| CINHAL  PsychINFO | (analyses, cost benefit OR analyses, cost-benefit OR analyses, cost-utility OR analyses, marginal OR analysis, cost benefit OR analysis, cost-benefit OR analysis, cost-effectiveness OR analysis, cost-utility OR analysis, marginal OR "benefits and costs" OR cost benefit OR cost benefit analyses OR cost benefit analysis OR cost benefit data OR cost effectiveness OR cost effectiveness analysis OR cost utility analysis OR cost-benefit analyses OR cost-benefit analysis OR cost-benefit data OR cost-effectiveness analysis OR cost-utility analyses OR cost-utility analysis OR "costs and benefits" OR data, cost-benefit OR economic evaluation OR economic evaluations OR effectiveness, cost OR evaluation, economic OR evaluations, economic OR marginal analyses OR marginal analysis OR cost analysis OR cost analyses OR cost-consequence OR cost consequence) | (persistent depression. OR depression, refractory OR depression, therapy-resistant OR depression, treatment resistant OR depressions, refractory OR depressions, therapy-resistant OR depressions, treatment resistant OR depressive disorder, treatment resistant OR depressive disorder, treatment-resistant OR depressive disorders, treatment-resistant OR disorder, treatment-resistant depressive OR disorders, treatment-resistant depressive OR refractory depression OR refractory depressions OR resistant depression, treatment OR resistant depressions, treatment OR therapy resistant depression OR therapy-resistant depression OR therapy-resistant depressions OR treatment resistant depression OR treatment resistant depressions OR treatment-resistant depressive disorder OR treatment-resistant depressive disorders) |
| NHSEED  HTA Cochrane | (analyses, cost benefit OR analyses, cost-benefit OR analyses, cost-utility OR analyses, marginal OR analysis, cost benefit OR analysis, cost-benefit OR analysis, cost-effectiveness OR analysis, cost-utility OR analysis, marginal OR "benefits and costs" OR cost benefit OR cost benefit analyses OR cost benefit analysis OR cost benefit data OR cost effectiveness OR cost effectiveness analysis OR cost utility analysis OR cost-benefit analyses OR cost-benefit analysis OR cost-benefit data OR cost-effectiveness analysis OR cost-utility analyses OR cost-utility analysis OR "costs and benefits" OR data, cost-benefit OR economic evaluation OR economic evaluations OR effectiveness, cost OR evaluation, economic OR evaluations, economic OR marginal analyses OR marginal analysis OR cost analysis OR cost analyses OR cost-consequence OR cost consequence) AND (MeSH DESCRIPTOR Depressive Disorder, Treatment-Resistant EXPLODE ALL TREES) | (persistent depression. OR depression, refractory OR depression, therapy-resistant OR depression, treatment resistant OR depressions, refractory OR depressions, therapy-resistant OR depressions, treatment resistant OR depressive disorder, treatment resistant OR depressive disorder, treatment-resistant OR depressive disorders, treatment-resistant OR disorder, treatment-resistant depressive OR disorders, treatment-resistant depressive OR refractory depression OR refractory depressions OR resistant depression, treatment OR resistant depressions, treatment OR therapy resistant depression OR therapy-resistant depression OR therapy-resistant depressions OR treatment resistant depression OR treatment resistant depressions OR treatment-resistant depressive disorder OR treatment-resistant depressive disorders) AND (MeSH DESCRIPTOR Cost-Benefit Analysis, Treatment-Resistant EXPLODE ALL TREES) |

**Table S2:** CHEC reporting items for included studies

| First author | Atlas | Desai | Edwards | Fitzgibbon | Galletly | Ghiasvand | Health Quality Ontario | Hollinghurst | HTA Calgary | Kessler | Knapp |
| --- | --- | --- | --- | --- | --- | --- | --- | --- | --- | --- | --- |
| Study design | Model | Model | Model | Model | Model | Model | Model | Trial | Model | Trial | Trial |
| Is a well-defined research question posed in answerable form? | Yes | Yes | Yes | Yes | Yes | Yes | Yes | Yes | Yes | Yes | Yes |
| Is the study population clearly described? | Yes | Yes | Yes | No | Yes | No | Yes | Yes | Yes | Yes | Yes |
| Are competing alternatives clearly described? | Yes | Yes | Yes | Yes | Yes | No | Yes | Yes | Yes | Yes | Yes |
| Is the actual perspective chosen appropriate? | Yes | Yes | Yes | Yes | Yes | Yes | Yes | Yes | Yes | Yes | Yes |
| Is the economic study design appropriate to the stated objective? | Yes | Yes | Yes | Yes | Yes | Yes | Yes | Yes | Yes | Yes | Yes |
| Is the chosen time horizon appropriate in order to include relevant costs and benefits? | Yes | Yes | Yes | Yes | Yes | Yes | Partly | Yes | Partly | Yes | Yes |
| Are all future costs and benefits discounted appropriately? | Yes | Yes | Yes | Yes | Yes | Yes | Yes | Yes | Yes | Yes | Yes |
| Are the structural assumptions and the validation methods of the model properly reported? | Yes | No | Yes | No | Yes | No | Yes | n/a | No | n/a | n/a |
| Are all important and relevant costs for each alternative identified? | Unclear | Yes | Yes | Yes | Yes | Yes | Yes | Yes | No | Yes | Yes |
| Are all costs measured appropriately in physical units? | Unclear | Yes | Yes | Yes | Yes | Yes | Yes | Yes | Yes | Yes | Yes |
| Are costs valued appropriately? | Yes | Yes | Yes | Yes | Unclear | Unclear | Yes | Yes | Yes | Yes | Yes |
| Are all important and relevant benefits for each alternative identified? | Yes | Yes | Yes | Yes | Yes | Unclear | Yes | Yes | Yes | Yes | Yes |
| Are all benefits measured appropriately? | Yes | Yes | Yes | Yes | Yes | No | Partly | Yes | Partly | Yes | Yes |
| Are benefits valued appropriately? | Yes | Yes | Yes | Yes | Yes | No | Yes | Yes | No | Yes | Yes |
| Is an appropriate incremental analysis of costs and benefits of alternatives performed? | Yes | Yes | Yes | Yes | Yes | No | Yes | Yes | Partly | Yes | Yes |
| Do the conclusions follow from the data reported? | Yes | Yes | Yes | Yes | Yes | Yes | Yes | Yes | Yes | Yes | Yes |
| Does the study discuss the generalizability of the results to other settings and patient/client groups? | No | Yes | No | Yes | No | No | Yes | Yes | No | Yes | Yes |
| Does the article indicate/report that there is no potential Conflict of Interest (COI) of study researcher(s) and funder(s)? | Yes | Conflict declared | Yes | Conflict declared | Yes | Yes | Yes | Yes | Yes | Yes | Yes |
| Are ethical and distributional issues discussed appropriately? | Yes | No | No | Yes | No | Yes | Yes | Yes | Yes | Yes | Yes |

| First author | Kozel | Lynch | Malone | McDonald | Morriss | Nguyen | Olgiati | Ross (2018) | Ross (2020) | Scott | Shearer |
| --- | --- | --- | --- | --- | --- | --- | --- | --- | --- | --- | --- |
| Study design | Model | Trial | Model | Trial | Trial | Model | Model | Model | Model | Trial | Trial |
| Is a well-defined research question posed in answerable form? | Yes | Yes | No | Yes | Yes | Yes | Yes | Yes | Yes | Yes | Yes |
| Is the study population clearly described? | Yes | Yes | No | Yes | Yes | No | Yes | Yes | Yes | Yes | Yes |
| Are competing alternatives clearly described? | Yes | Yes | Yes | Yes | Yes | Yes | No | Yes | Yes | Yes | Yes |
| Is the actual perspective chosen appropriate? | Yes | Yes | Yes | Yes | Yes | Yes | Yes | Yes | Yes | Yes | Yes |
| Is the economic study design appropriate to the stated objective? | Yes | Yes | Unclear | No | Yes | Yes | Yes | Yes | Yes | Yes | Yes |
| Is the chosen time horizon appropriate in order to include relevant costs and benefits? | Partly | Yes | Partly | Yes | Yes | Yes | Partly | Yes | Yes | Yes | Yes |
| Are all future costs and benefits discounted appropriately? | Yes | Yes | Yes | Yes | Yes | Yes | Yes | Yes | Yes | Yes | Yes |
| Are the structural assumptions and the validation methods of the model properly reported? | Yes | n/a | No | n/a | n/a | No | No | Yes | Yes | N/A | N/A |
| Are all important and relevant costs for each alternative identified? | Unclear | Yes | Unclear | Yes | Yes | Yes | No | Unclear | Unclear | Yes | Yes |
| Are all costs measured appropriately in physical units? | Unclear | Yes | Unclear | Yes | Yes | Yes | Yes | Yes | Yes | Yes | Yes |
| Are costs valued appropriately? | Unclear | Yes | Yes | Unclear | Yes | Yes | Yes | Yes | Yes | Yes | Yes |
| Are all important and relevant benefits for each alternative identified? | Yes | Yes | Yes | Yes | Yes | Yes | Yes | Yes | Yes | Yes | Yes |
| Are all benefits measured appropriately? | Yes | Yes | Partly | Yes | Yes | Partly | Yes | Yes | Yes | Yes | Yes |
| Are benefits valued appropriately? | No | Yes | Yes | Yes | Unclear | Yes | No | Yes | Yes | N/A | Yes |
| Is an appropriate incremental analysis of costs and benefits of alternatives performed? | Yes | Yes | Yes | No | Yes | Yes | Yes | Yes | Yes | Yes | Yes |
| Do the conclusions follow from the data reported? | Yes | Yes | Yes | Yes | Yes | Yes | Yes | Yes | Yes | Yes | Yes |
| Does the study discuss the generalizability of the results to other settings and patient/client groups? | Yes | Yes | No | No | Yes | Yes | Yes | Yes | Yes | No | Yes |
| Does the article indicate/report that there is no potential Conflict of Interest (COI) of study researcher(s) and funder(s)? | Yes | Yes | Conflict declared | No | Yes | No | Conflict declared | Yes | Yes | Yes | No |
| Are ethical and distributional issues discussed appropriately? | No | Yes | Yes | No | No | No | No | Yes | Yes | No | Yes |

| First author | Simon | Simpson | Town | Vallejo-Torres | Wang | Wiles | Xie | Young | Zhao |
| --- | --- | --- | --- | --- | --- | --- | --- | --- | --- |
| Study design | Trial | Model | Trial | Model | Model | Trial | Model | Model | Model |
| Is a well-defined research question posed in answerable form? | Yes | Yes | Yes | Yes | Yes | Yes | Yes | Yes | Yes |
| Is the study population clearly described? | Yes | Yes | Yes | Yes | Yes | Yes | Yes | Yes | No |
| Are competing alternatives clearly described? | Yes | Yes | Yes | Yes | Yes | Yes | Yes | Yes | Yes |
| Is the actual perspective chosen appropriate? | Yes | Yes | Yes | Yes | Yes | Yes | Yes | Yes | Yes |
| Is the economic study design appropriate to the stated objective? | Yes | Yes | Yes | Yes | Yes | Yes | Yes | Yes | Yes |
| Is the chosen time horizon appropriate in order to include relevant costs and benefits? | Yes | Yes | Yes | Yes | Yes | Yes | Partly | Yes | Yes |
| Are all future costs and benefits discounted appropriately? | Yes | Yes | Yes | Yes | Yes | Yes | Yes | Yes | Yes |
| Are the structural assumptions and the validation methods of the model properly reported? | N/A | No | N/A | Yes | Yes | N/A | Yes | Yes | No |
| Are all important and relevant costs for each alternative identified? | Yes | Yes | Yes | Yes | Yes | Yes | Yes | Yes | Yes |
| Are all costs measured appropriately in physical units? | Yes | Yes | Yes | Yes | Yes | Yes | Yes | Yes | Yes |
| Are costs valued appropriately? | Yes | Yes | Yes | Yes | Yes | Yes | Yes | Yes | Yes |
| Are all important and relevant benefits for each alternative identified? | Yes | Yes | Yes | Yes | Yes | Yes | Yes | Yes | Yes |
| Are all benefits measured appropriately? | Unclear | Yes | Yes | Yes | Yes | Yes | Yes | Partly | No |
| Are benefits valued appropriately? | N/A | Yes | Yes | Yes | Yes | Yes | Yes | Yes | Yes |
| Is an appropriate incremental analysis of costs and benefits of alternatives performed? | Yes | Partly | Yes | Yes | Yes | Yes | Yes | Yes | Yes |
| Do the conclusions follow from the data reported? | Yes | Yes | Yes | Yes | Yes | Yes | Yes | Yes | Yes |
| Does the study discuss the generalizability of the results to other settings and patient/client groups? | Partly | Yes | No | Yes | No | Yes | Yes | Partly | Yes |
| Does the article indicate/report that there is no potential Conflict of Interest (COI) of study researcher(s) and funder(s)? | No | Conflict declared | Yes | Yes | Conflict declared | Yes | Yes | No | Yes |
| Are ethical and distributional issues discussed appropriately? | No | No | No | No | No | Yes | No | No | No |

**Table S3**: Primary clinical and economic outcome measures used in the studies. Some model-based studies synthesised outcomes from multiple different sources, with unspecified outcome measures.

| Author, year | Primary clinical outcome | Primary economic outcome measure |
| --- | --- | --- |
| Atlas, 2020 | Remission/ Response (MADRS, QIDS-SR) | QALYs (EQ-5D-3L) |
| Desai, 2021 | Remission/ response (MADRS) | Cost per remitter |
| Edwards, 2013 | Remission/ response (MADRS) | QALYs (EQ-5D-3L) |
| Fitzgibbon, 2020 | Remission (MADRS, HAM-D, QIDS-SR) | QALYs (EQ-5D-3L; SG) |
| Galletly, 2014 | Response/ remission (HAM-D) | QALYs (EQ-5D-3L) |
| Ghiasvand, 2016 | Remission | QALYs (SF-6D) |
| Health Quality Ontario, 2016 | Remission/ response (MADR-S, HAM-D) | QALYs (EQ-5D-3L) |
| Hollinghurst, 2014 | Response (BDI-II) | QALYs (EQ-5D-3L; SF-6D) |
| HTA Calgary , 2014 | Remission/ response | QALYs (SF-6D; SG) |
| Kessler, 2018 | Response (BDI-II) | QALYs (EQ-5D-3L) |
| Knapp, 2008 | Depressive symptoms/ Remission (HAM-D) | HAM-D |
| Kozel, 2004 | Response (HAM-D) | QALYs (HUI-3) |
| Lynch, 2018 | Response (HAM-D) | QALYs (EQ-5D-3L) |
| Malone, 2007 | Remission (MADRS, HAM-D) | Cost per remission |
| McDonald, 1998 | Maintenance of response/ relapse (MADRS; BDI; GAF) | N/A (cost consequence analysis) |
| Morriss, 2016 | Response (HAM-D) | QALYs (EQ-5D-3L) |
| Nguyen, 2015 | Remission/ response (HAM-D) | QALYs (EQ-5D-3L) |
| Olgiati, 2013 | Remission | QALYs (SG) |
| Ross, 2018 | Remission/ response/ relapse | QALYs (EQ-5D-3L) |
| Ross, 2020 | Remission/ response | QALYs (EQ-5D-3L) |
| Scott, 2003 | Relapse (HAM-D) | Cost per relapse prevented |
| Shearer, 2019 | Depressive symptoms (HAM-D) | QALYs (EQ-5D-3L) |
| Simon, 2001 | Depression free days (SCL-90) | Cost per depression free day |
| Simpson, 2009 | Depressive symptoms (MADRS) | QALYs (SG; McSad) |
| Town, 2020 | Depressive symptoms (HAM-D) | QALYs (SF-6D) |
| Vallejo-Torres, 2015 | Response / remission (HAM-D) | QALYs (EQ-5D-3L; McSad) |
| Wang, 2019 | Response / remission (MADRS) | QALYs (EQ-5D-3L) |
| Wiles, 2016 | Depressive symptoms (BDI-II) | QALYs (EQ-5D-3L) |
| Xie, 2019 | Remission/ response (MADR-S, HAM-D) | QALYs (EQ-5D-3L) |
| Young, 2017 | Response/ remission/ recovery | QALYs (EQ-5D-3L) |
| Zhao, 2018 | Remission (HAM-D, MADRS) | QALYs (EQ-5D-3L) |

**Table S4:** Health state utilities and sources used in model-based CUAs

| Author, year | TRD population/ definition | Utility values | Source of utility data |
| --- | --- | --- | --- |
| Atlas, 2020 | Inadequate response to ≥2 AD treatments | Remission: 0.85 Mild-moderate: 0.68 Severe: 0.50 | Remission - weighted US average EQ-5D Depressed states: Janiack *et al.* (1) |
| Edwards, 2013 | Inadequate response to ≥2 AD treatments | Remission: 0.85 Response: 0.72 Non-response: 0.58 | Sapin *et al.*(2) |
| Fitzgibbon, 2020 | Not explicitly defined | Remission: 0.80 ECT treatment: 0.55 rTMS treatment:0.63 Maintenance treatment - 0.72 Severe depression - 0.3 | Sapin *et al.*(2)  Revicki and Wood (3)  Zhao *et al.* (4) |
| Galletly, 2014 | Inadequate response to ≥2 AD treatments | Remission: 0.86 Partial remission: 0.71 No response: 0.52 | Utilities: Hawthorne *et al.* (5) Disutilities: Sullivan *et al.*(6) |
| Ghiasvand, 2016 | Not explicitly defined | Utilities appear to be incorrectly calculated in this report | McLoughlin *et al.* (7) |
| Health Quality Ontario, 2016 | Inadequate response to ≥2 AD treatments | No response: 0.58 Partial response: 0.72 Remission: 0.85 | Sapin *et al.*(2) |
| HTA Calgary , 2014 | Inadequate response to ≥2 AD treatments | Relapse/No response: 0.3 Response: 0.73 Remission: 0.83 | Revicki and Wood (3)  McLoughlin *et al.* (7) |
| Kozel, 2004 | Not explicitly defined | Depressed patients: 0.25 Recovered depressed patients: 0.91 | Feeny *et al.* (8) |
| Nguyen, 2015 | Inadequate response to ≥2 AD treatments | Response: 0.71 Remission: 0.86 No response: 0.52 | Utilities: Hawthorne *et al.* (5) Disutilities: Sullivan *et al.*(6) |
| Olgiati, 2013 | Inadequate response to single AD treatment | Acute depression / relapse: 0.4 Remission: 0.9 Treatment disutility: 0.04 | Revicki and Wood (3) |
| Ross, 2018 | Inadequate response to ≥2 AD treatments | Remission: 0.85 Response: 0.72 Nonresponse, relapse, initiation: 0.58 | Sapin *et al.*(2) |
| Ross, 2020 | Inadequate response to ≥2 AD treatments | Remission: 0.85 Response: 0.72 Nonresponse, relapse, initiation: 0.58 | Sapin *et al.*(2) |
| Simpson, 2009 | Inadequate response to 1-4 AD treatment | Remission: 0.83 Mild: 0.73 Moderate: 0.63 Severe: 0.3 In-hospital failure: 0.09 | Revicki and Wood (3) |
| Vallejo-Torres, 2015 | Not explicitly defined | **McSad** Acute Tx 0.09 Continuation Tx 0.59 Stable 0.79 Moderate depression 0.32 Severe depression 0.09  **EQ-5D-3L** Acute Tx 0.519 Continuation Tx 0.645 Stable 0.759 Moderate depression 0.558 Severe depression 0.337 | McSad: (9)  EQ-5D-3L: (10) |
| Wang, 2019 | Inadequate response to ≥2 AD treatments | Full remission 0.86 Partial remission 0.74 In episode/ relapse 0.44 | Sapin *et al.*(2) |
| Xie, 2019 | Inadequate response to ≥2 AD treatments | No response: 0.58 Partial response: 0.72 Remission: 0.85 | Sapin *et al.*(2) |
| Young, 2017 | Inadequate response to ≥2 AD treatments | **Acute (0-8) utilities** Depression at baseline 0.54 Remission 0.85 Response without remission 0.76 No response 0.56  **Maintenance utilities:** Remission 0.85 Response without remission 0.76 Relapse 0.56 Recovery 0.85 Recurrence 0.56  Disutilities for adverse events short & long term ranged from 0 (dizziness, sweating, dry mouth), to 0.129 (insomnia) | Utilities: Montgomery *et al.* (11)  Disutilities: Sullivan *et al.*(6) |
| Zhao, 2018 | Not explicitly defined | Treatment-resistant depression 0.56 Remission after acute treatment 0.80 Remission after maintenance therapy 0.84 No remission 0.55 Relapse 0.63 Severe depression§ 0.30 | Hospital chart data |

1. Janicak PG, Dunner DL, Aaronson ST, Carpenter LL, Boyadjis TA, Brock DG, et al. Transcranial magnetic stimulation (TMS) for major depression: a multisite, naturalistic, observational study of quality of life outcome measures in clinical practice. CNS Spectr. 2013;18(6):322-32.

2. Sapin C, Fantino B, Nowicki ML, Kind P. Usefulness of EQ-5D in assessing health status in primary care patients with major depressive disorder. Health Qual Life Outcomes. 2004;2:20.

3. Revicki DA, Wood M. Patient-assigned health state utilities for depression-related outcomes: differences by depression severity and antidepressant medications. J Affect Disord. 1998;48(1):25-36.

4. Zhao YJ, Tor PC, Khoo AL, Teng M, Lim BP, Mok YM. Cost-Effectiveness Modeling of Repetitive Transcranial Magnetic Stimulation Compared to Electroconvulsive Therapy for Treatment-Resistant Depression in Singapore. Neuromodulation. 2018;21(4):376-82.

5. Hawthorne G, Cheok F, Goldney R, Fisher L. The excess cost of depression in South Australia: a population-based study. Aust N Z J Psychiatry. 2003;37(3):362-73.

6. Sullivan PW, Valuck R, Saseen J, MacFall HM. A comparison of the direct costs and cost effectiveness of serotonin reuptake inhibitors and associated adverse drug reactions. CNS Drugs. 2004;18(13):911-32.

7. McLoughlin DM, Mogg A, Eranti S, Pluck G, Purvis R, Edwards D, et al. The clinical effectiveness and cost of repetitive transcranial magnetic stimulation versus electroconvulsive therapy in severe depression: a multicentre pragmatic randomised controlled trial and economic analysis. Health Technol Assess. 2007;11(24):1-54.

8. Feeny D, Furlong W, Torrance GW, Goldsmith CH, Zhu Z, DePauw S, et al. Multiattribute and Single-Attribute Utility Functions for the Health Utilities Index Mark 3 System. Medical Care. 2002;40(2):113-28.

9. Bennett KJ, Torrance GW, Boyle MH, Guscott R, Moran LA. Development and Testing of a Utility Measure for Major, Unipolar Depression (McSad). Quality of Life Research. 2000;9(1):109-20.

10. Mann R, Gilbody S, Richards D. Putting the 'Q' in depression QALYs: a comparison of utility measurement using EQ-5D and SF-6D health related quality of life measures. Soc Psychiatry Psychiatr Epidemiol. 2009;44(7):569-78.

11. Montgomery SA, Nielsen RZ, Poulsen LH, Häggström L. A randomised, double-blind study in adults with major depressive disorder with an inadequate response to a single course of selective serotonin reuptake inhibitor or serotonin–noradrenaline reuptake inhibitor treatment switched to vortioxetine or agomelatine. Human Psychopharmacology: Clinical and Experimental. 2014;29(5):470-82.
